# Universal immunotherapeutic strategy for hepatocellular carcinoma with exosome vaccines that engage adaptive and innate immune responses

**DOI:** 10.1186/s13045-022-01266-8

**Published:** 2022-04-29

**Authors:** Bingfeng Zuo, Yang Zhang, Kangjie Zhao, Li Wu, Han Qi, Rong Yang, Xianjun Gao, Mengyuan Geng, Yingjie Wu, Renwei Jing, Qibing Zhou, Yiqi Seow, HaiFang Yin

**Affiliations:** 1 The Province and Ministry Co-Sponsored Collaborative Innovation Center for Medical Epigenetics and Key Laboratory of Immune Microenvironment and Disease (Ministry of Education) and School of Medical Technology and School of Basic Medical Sciences, Tianjin Medical University, Qixiangtai Road, Heping District, Tianjin, 300070 China; 2 Department of Nanomedicine and Biopharmaceuticals, National Engineering Research Center for Nanomedicine, Huazhong University of Science and Technology, Wuhan, 430074 Hubei Province China; 3 Institute of Bioengineering and Bioimaging, 31 Biopolis Way, Singapore, 138669 Singapore; 4 Institute of Molecular and Cell Biology, 61 Biopolis Way, Singapore, 138668 Singapore

**Keywords:** Exosome, Hepatocellular carcinoma, Personalized immunotherapy, Adaptive and innate immunity

## Abstract

**Background:**

Personalized immunotherapy utilizing cancer vaccines tailored to the tumors of individual patients holds promise for tumors with high genetic heterogeneity, potentially enabling eradication of the tumor in its entirety.

**Methods:**

Here, we demonstrate a general strategy for biological nanovaccines that trigger tailored tumor-specific immune responses for hepatocellular carcinoma (HCC). Dendritic cell (DC)-derived exosomes (DEX) are painted with a HCC-targeting peptide (P47-P), an α-fetoprotein epitope (AFP212-A2) and a functional domain of high mobility group nucleosome-binding protein 1 (N1ND-N), an immunoadjuvant for DC recruitment and activation, via an exosomal anchor peptide to form a “trigger” DEX vaccine (DEX_P&A2&N_).

**Results:**

DEX_P&A2&N_ specifically promoted recruitment, accumulation and activation of DCs in mice with orthotopic HCC tumor, resulting in enhanced cross-presentation of tumor neoantigens and de novo T cell response. DEX_P&A2&N_ elicited significant tumor retardation and tumor-specific immune responses in HCC mice with large tumor burdens. Importantly, tumor eradication was achieved in orthotopic HCC mice when antigenic AFP peptide was replaced with the full-length AFP (A) to form DEX_P&A&N_. Supplementation of Fms-related tyrosine kinase 3 ligand greatly augmented the antitumor immunity of DEX_P&A&N_ by increasing immunological memory against tumor re-challenge in orthotopic HCC mice. Depletion of T cells, cross-presenting DCs and other innate immune cells abrogated the functionality of DEX_P&A&N_.

**Conclusions:**

These findings demonstrate the capacity of universal DEX vaccines to induce tumor-specific immune responses by triggering an immune response tailored to the tumors of each individual, thus presenting a generalizable approach for personalized immunotherapy of HCC, by extension of other tumors, without the need to identify tumor antigens.

**Supplementary Information:**

The online version contains supplementary material available at 10.1186/s13045-022-01266-8.

## Introduction

Hepatocellular carcinoma (HCC) is one of the most lethal malignancies worldwide, particularly in the Asia–Pacific region [1]; however, there is no effective non-surgical treatment available in the clinic. Although immunotherapy has emerged as a mainstream therapeutic modality for HCC, early therapeutic vaccination strategies that focused on tumor-associated antigens (TAAs) alone were largely unsuccessful at delivering clinically effective antitumor immune response due to tumor heterogeneity [2]. This highlights the importance of developing cancer vaccines with enhanced tumor specificity and immunogenicity [3]. Recently, neoantigen-based peptide, mRNA, DNA or dendritic cell (DC) personalized vaccines have been investigated in melanoma, non-small cell lung cancer and renal cancer, with a number of promising ongoing clinical trials [3, 4]. These individualized vaccines were designed to trigger de novo antigen-specific T cell response against tumor neoantigens. However, high tumor heterogeneity for HCC has resulted in difficulty of neoantigen identification [5] and hence poor response. In addition, the paucity of known neoepitopes presented in human leukocyte antigen class I complexes for nonsynonymous HCC mutations in patients raised the concern that neoantigen-specific T cells alone might not be adequate to exert cytotoxic killing in HCC [6].

Approaches that target the tumors of individual patients and trigger tumor-specific immune responses without the need to identify each tumor antigen will unlock the full therapeutic potential of personalized immunotherapy. We believe that a strategy enabling targeted recruitment and activation of endogenous DCs to tumor sites can stimulate cross-presentation of tumor antigens and allow generation of tumor-specific immune responses against neoantigens within the tumor, thus achieving the goal of personalized immunotherapy without the need and regulatory nightmare of developing individualized products for each patient. To this end, we painted DC-derived exosomes (DEX), acellular vaccines extensively employed for tumor immunotherapy with high biocompatibility and low or no immunogenicity [7–9], with the functional domain of high mobility group nucleosome-binding protein 1 (HMGN1) (N1ND-N), an immunoadjuvant capable of promoting recruitment and activation of DCs [10, 11], a HCC-targeting peptide-P47 (P) [12] and an α-fetoprotein epitope (AFP212 -A2) [13] via our previously identified exosomal anchor peptide (CP05) [14] to form a designer vaccine DEX_P&A2&N_. The rationally designed DEX_P&A2&N_ would enable tumor-targeted delivery of N1ND and promote N1ND-mediated recruitment and activation of endogenous DCs in tumor in the presence of HCC antigens, resulting in cross-presentation of tumor antigens and induction of tumor-specific T cell responses.

Here, we demonstrate that DEX_P&A2&N_ resulted in recruitment, specific accumulation and activation of cross-presenting CD103^+^CD11c^+^ and CD8α^+^CD11c^+^ DCs [15] in tumor of orthotopic HCC mice after intravenous administration. Recruited DCs cross-presented tumor antigens and triggered antigen-specific de novo immune responses, and significant tumor retardation in orthotopic HCC mice bearing large established tumors. Importantly, complete tumor eradication was achieved in orthotopic HCC mice when AFP212 was replaced with the full-length AFP [16] to form DEX_P47&AFP&N1ND_ (DEX_P&A&N_) vaccines. Incorporation of Fms-related tyrosine kinase 3 ligand (Flt3L), a major regulator for DC development [17, 18], to the DEX_P&A&N_ regime further augmented the antitumor immunogenicity, elicited long-lived protective T cell memory against tumor re-challenge and resulted in tumor eradication in a majority of mice with established orthotopic HCC tumors. These findings demonstrate the capacity of universal DEX vaccines to induce tumor-specific immune responses by triggering a tailored immune response to the tumors of each individual, and also present a generalizable approach for personalized immunotherapy of HCC, by extension of other tumors, without the need to identify tumor antigens.

## Results

### ***Tumor-targeted DEX***_***P&A2&N***_*** promote DC recruitment and activation in tumor***

**Fig. 1:**
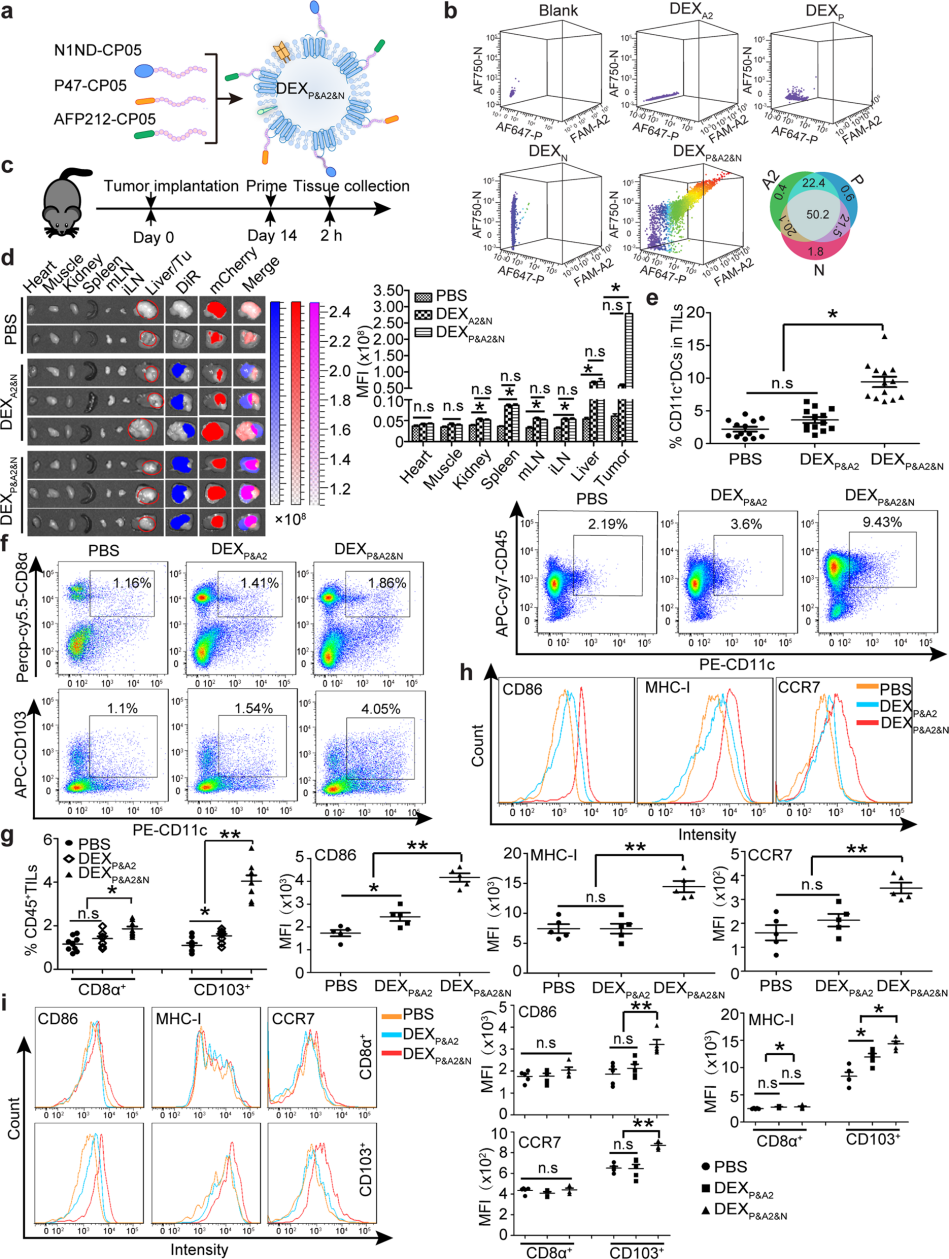
Evaluation of DEX_P&A2&N_’s tumor-targeting, DC-recruiting and DC-activating ability in orthotopic mCherry-expressing HCC mice. **a** Schematic illustration for designer DEX vaccine-DEX_P&A2&N_. P-P47; A2-AFP212; N-N1ND. DEX refers to DC-derived exosomes. **b** Flow cytometric analysis to assess the simultaneous binding efficiency of three moieties on DEX (*n* = 4). Diagram for dosing regimen (**c**) and tissue distribution and quantitative analysis of labeled DEX_P&A2&N_ (**d**) in day-14 orthotopic HCC mice bearing mCherry-expressing tumors. DiR-labeled DEX_A2&N_ (*n* = 9), DEX_P&A2&N_ (*n* = 9) (80 μg/mouse) or PBS (*n* = 4) were injected into day-14 orthotopic HCC mice bearing mCherry-expressing tumors intravenously, and tissues were harvested 2 h after injection (one-way ANOVA post hoc Student–Newman–Keuls test was used except for liver and tumor in which one-way ANOVA on ranks was used). mLN-mesenteric lymph node; iLN-inguinal lymph node; Tu-tumor. **e **Flow cytometric and quantitative analysis of CD11c^+^ DCs in tumor-infiltrating lymphocytes (TILs) from HCC mice bearing mCherry-expressing tumors (*n* = 13; one-way ANOVA post hoc Student–Newman–Keuls test). Flow cytometric (**f**) and quantitative analysis (**g**) of CD103^+^CD11c^+^ (one-way ANOVA on ranks) and CD8α^+^ CD11c^+^ DCs (one-way ANOVA post hoc Student–Newman–Keuls test) in TILs from HCC mice (*n* = 9). **h** Flow cytometric and quantitative analysis of surface protein markers on CD11c^+^ DCs from tumors of orthotopic HCC mice treated with DEX_P&A2_ or DEX_P&A2&N_ (*n* = 5; one-way ANOVA post hoc Student–Newman–Keuls test). **i** Flow cytometric and quantitative analysis of surface protein markers on CD103^+^CD11c^+^ and CD8α^+^CD11c^+^ DCs from tumors of orthotopic HCC mice treated with DEX_P&A2_ or DEX_P&A2&N_ (*n* = 5; one-way ANOVA post hoc Student–Newman–Keuls test). *p < 0.05, **p < 0.001; n.s, not significant

To assemble a designer DEX vaccine tailored to the tumor of each individual, we loaded DEX, characterized with a sauce-cup shape and mean diameter of 117 nm and expression of exosomal biomarkers [19] (Additional file 1: Figure S1a-c), with the HCC-targeting peptide P47 (P) [12], antigenic epitope AFP212 (A2) [13] and N1ND (N) [10] via the exosomal anchor peptide CP05 [14] to form DEX_P&A2&N_ (Fig. 1a), with a triple loading efficiency of 50.2% (Fig. 1b). Loading of functional moieties did not alter the morphology and size of DEX (Additional file 1: Figure S1a and S1b). Significantly elevated fluorescence signals were observed in tumor 2 h after DiR-labeled DEX_P&A2&N_ were intravenously administered into day-14 orthotopic HCC mice bearing mCherry-expressing tumors, compared to DEX_A2&N_ and PBS controls (Fig. 1c, d), confirming that P47 enables targeted delivery of DEX_P&A2&N_ to tumor. Notably, substantially higher fluorescence was also detected in spleen, mesenteric and inguinal lymph nodes from mice treated with DEX_P&A2&N_ and DEX_A2&N_ than PBS controls, indicating an intrinsic tropism of DEX (Fig. 1d). Strikingly, a significant rise in the number of CD11c^+^ DCs including CD11b^+^CD11c^+^, a potent driver of CD4^+^ helper T cell response [20], and CD11b^−^ CD11c^+^ DCs [10] (Fig. 1e; Additional file 1: S1d), particularly migratory CD103^+^CD11c^+^ and resident CD8α^+^CD11c^+^ DCs, two major DC subsets that excel in the priming and cross-presentation of cell-associated antigens to CD8^+^ T cells [15, 20, 21], was found in tumor-infiltrating lymphocytes from orthotopic HCC mice treated with DEX_P&A2&N_ compared to DEX_P&A2_ and PBS controls (Fig. 1f, g). Elevated expression of major histocompatibility complex I (MHC I), CD86 and CCR7, markers for DC activation [22–24], was also detected in CD103^+^CD11c^+^ DCs, to a lesser extent with resident cross-presenting CD8α^+^CD11c^+^ DCs (Fig. 1h, i). These results demonstrated DEX_P&A2&N_-mediated targeted recruitment and activation of DCs, particularly cross-presenting CD103^+^CD11c^+^ DCs to tumor sites in orthotopic HCC mice.

### ***DEX***_***P&A2&N***_*** boost DC uptake and cross-presentation of neoantigens***

**Fig. 2:**
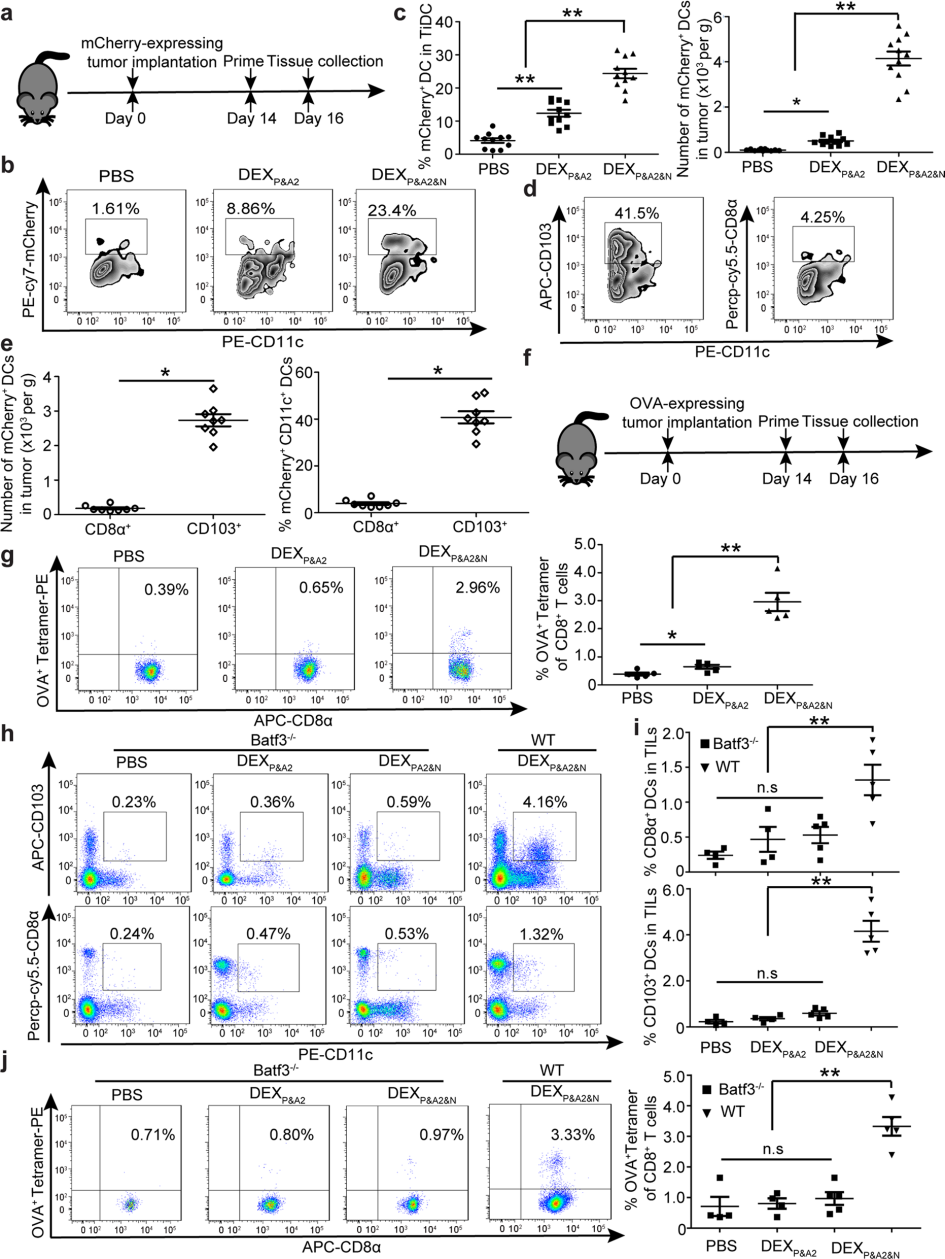
Investigation of DEX_P&A2&N_ promoting DC uptake and cross-presentation of tumor neoantigens in orthotopic HCC mice. **a** Diagram for dosing regimen of DEX_P&A2&N_. DEX_P&A2_ or DEX_P&A2&N_ (80 μg/mouse) were injected into day-14 orthotopic HCC mice bearing mCherry-expressing tumors intravenously, and tissues were harvested 2 days after injection. Flow cytometric (**b**) and quantitative analysis (**c**) of mCherry^+^CD11c^+^ DCs from tumor of orthotopic HCC mice treated with DEX_P&A2_ or DEX_P&A2&N_ (*n* = 11) (One-way ANOVA on ranks was used for the number and one-way ANOVA post hoc Student–Newman–Keuls test was applied for the percent analysis). TiDC: tumor-infiltrating DC. Flow cytometric (**d**) and quantitative analysis (**e**) of mCherry^+^CD103^+^CD11c^+^ or CD8α^+^CD11c^+^ DCs from tumor of orthotopic HCC mice treated with DEX_P&A2&N_ (*n* = 8; Mann–Whitney rank-sum test). **f** Diagram for dosing regimen of DEX_P&A2&N_. DEX_P&A2_ or DEX_P&A2&N_ (80 μg/mouse) were injected into day-14 orthotopic HCC mice bearing OVA-expressing tumors intravenously, and tissues were harvested 2 days after injection. **g** Flow cytometric and quantitative analysis of OVA^+^ tetramer T cells from splenocytes of orthotopic HCC mice treated with DEX_P&A2_ or DEX_P&A2&N_ (*n* = 5; one-way ANOVA on ranks). Flow cytometric (**h**) and quantitative analysis (**i**) of CD103^+^CD11c^+^ or CD8α^+^CD11c^+^ DCs from TILs of orthotopic Batf3^−/−^ HCC mice treated with PBS or DEX_P&A2_ (*n* = 4) or DEX_P&A2&N_ (*n* = 5) and orthotopic wild-type (WT) HCC mice treated with DEX_P&A2&N_ (*n* = 5) (one-way ANOVA on ranks). **j** Flow cytometric and quantitative analysis of OVA^+^ tetramer T cells from tumor of orthotopic Batf3^−/−^ HCC mice treated with PBS or DEX_P&A2_ (*n* = 4) or DEX_P&A2&N_ (*n* = 5) and orthotopic wild-type (WT) HCC mice treated with DEX_P&A2&N_ (*n* = 5) (one-way ANOVA post hoc Student–Newman–Keuls test). *p < 0.05, **p < 0.001; n.s, not significant

To examine the capability of recruited DCs to internalize and cross-present tumor neoantigens, we intravenously administered DEX_P&A2&N_ into day-14 orthotopic HCC mice bearing mCherry-expressing tumors (Fig. 2a). Significantly elevated tumor-associated mCherry^+^ fluorescent signals were observed in CD11c^+^ DCs from DEX_P&A2&N_-treated mice compared to DEX_P&A2_ and PBS controls (Fig. 2b, c), primarily in CD11b^−^CD11c^+^ DCs (Additional file 1: Figure S2a and S2b), demonstrating the superior cross-presenting ability of these DCs [25, 26]. CD103^+^CD11c^+^ DCs also outnumbered CD8α^+^CD11c^+^ DCs in tumor tissues of DEX_P&A2&N_-treated orthotopic HCC mice (Fig. 2d, e), indicating the stronger capacity of CD103^+^CD11c^+^ DCs to internalize and cross-present cellular neoantigens. Consistently, significantly increased numbers of mCherry^+^CD103^+^CD11c^+^ DCs were also found in spleen from DEX_P&A2&N_-treated mice (Additional file 1: Figure S2c), demonstrating the homing capacity of activated DCs to regional lymph nodes, a critical functional parameter for activated DCs [27]. To evaluate de novo host antitumor responses toward non-AFP antigens, we employed the ovalbumin (OVA)-expressing HCC model to directly assess the contribution of host immunity by detecting anti-OVA immune responses [28]. Substantially greater T cell responses specific for the SIINFEKL OVA peptide were detected in OVA-expressing HCC tumors from mice treated with DEX_P&A2&N_ than responses observed in DEX_P&A2_- or PBS-treated mice (Fig. 2f, g), suggesting that recruited DCs cross-presented OVA epitope and primed de novo T cell responses. As Batf3 is an important transcription factor required for development of cross-presenting CD103^+^CD11c^+^ and CD8α^+^CD11c^+^ DCs in mice [29, 30], to verify the role of cross-presenting DCs in the functionality of DEX_P&A2&N_, we intravenously administered DEX_P&A2&N_ to orthotopic Batf3^−/−^ HCC mice bearing OVA-expressing tumors. As expected, a significant decline in the number of CD11c^+^ DCs (Additional file 1: Figure S2d), particularly CD103^+^CD11c^+^ and CD8α^+^CD11c^+^ DCs, was observed in tumor-infiltrating lymphocytes of DEX_P&A2&N_-treated Batf3^−/−^ compared to wild-type mice (Fig. 2h, i). Corroborating with decreased numbers of DCs, OVA- and AFP-specific T cell responses were also significantly compromised in DEX_P&A2&N_-treated Batf3^−/−^ compared to wild-type mice (Fig. 2j; Additional file 1: S2e), with no inhibition on tumor growth (Additional file 1: Figure S2f), supporting the conclusion that cross-presenting DCs are primarily responsible for the uptake and cross-presentation of tumor neoantigens.

### ***DEX***_***P&A2&N***_*** elicit ***de novo*** immune responses and tumor suppression***

**Fig. 3:**
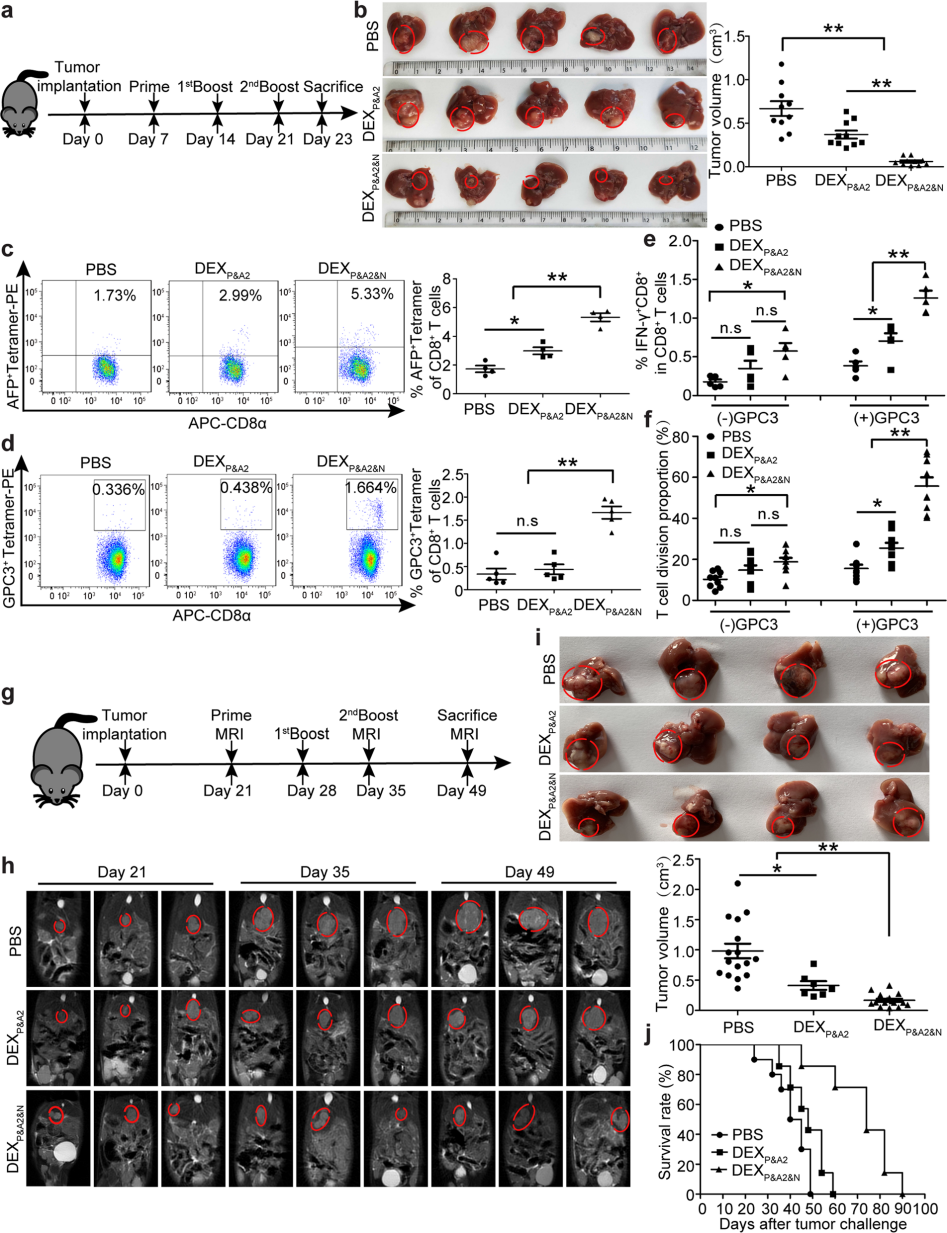
Antitumor effects of DEX_P&A2&N_ in orthotopic HCC mice. **a** Diagram for dosing regimen of DEX_P&A2&N_. DEX_P&A2_ or DEX_P&A2&N_ (80 μg/mouse) were injected into day-7 orthotopic HCC mice intravenously three times weekly, and tissues were harvested 2 days after last injection. **b** Measurement of tumor volume in day-7 orthotopic HCC mice treated with DEX_P&A2_ or DEX_P&A2&N_ (*n* = 10; one-way ANOVA on ranks). **c** Flow cytometric and quantitative analysis of AFP^+^ tetramer T cells from splenocytes of orthotopic HCC mice treated with DEX_P&A2_ or DEX_P&A2&N_ (*n* = 4; one-way ANOVA post hoc Student–Newman–Keuls test). **d** Flow cytometric and quantitative analysis of GPC3^+^ tetramer T cells from splenocytes of orthotopic HCC mice treated with DEX_P&A2_ or DEX_P&A2&N_ (*n* = 5; one-way ANOVA post hoc Student–Newman–Keuls test). Flow cytometric and quantitative analysis of IFN-γ^+^CD8^+^ T cells (**e**) (*n* = 5; one-way ANOVA post hoc Student–Newman–Keuls test) and T cell division (**f**) (*n* = 9; one-way ANOVA on ranks) in splenocytes of orthotopic HCC mice treated with DEX_P&A2_ or DEX_P&A2&N_, followed by in vitro stimulation of GPC3 epitopes. **g** Diagram for dosing regimen of DEX_P&A2&N_. DEX_P&A2_ or DEX_P&A2&N_ (120 μg/mouse) were injected into day-21 orthotopic HCC mice bearing large established tumors intravenously three times weekly, and tissues were harvested 2 weeks after last injection. **h** MRI monitoring of tumor growth in day-21 orthotopic HCC mice bearing large established tumors at different time points. **i** Assessment of tumor size in orthotopic HCC mice bearing large established tumors treated with PBS (*n* = 16), DEX_P&A2_ (*n* = 7) or DEX_P&A2&N_ (*n* = 16) at 28 days after initial treatment (one-way ANOVA on ranks). **j** Survival rate of orthotopic HCC mice treated with PBS (*n* = 9), DEX_P&A2_ or DEX_P&A2&N_ (*n* = 8). *p < 0.05, **p < 0.001; n.s, not significant

To assess the antitumor immunity, we administered DEX_P&A2&N_ intravenously in day-7 orthotopic HCC mice and observed a robust tumor regression in mice treated with DEX_P&A2&N_ (Fig. 3a, b). In agreement with effective tumor suppression, significantly larger numbers of T cells specific for the GSMLNEHVM AFP peptide were detected in DEX_P&A2&N_-treated mice than DEX_P&A2_ and PBS controls (Fig. 3c) [13]. Importantly, greater T cell responses specific for AMFKNNYPSL glypican3 (GPC3) peptide [31] were observed in DEX_P&A2&N_-treated mice than response detected in DEX_P&A2_ and PBS groups (Fig. 3d–f), indicating the generation of de novo T cell responses to epitopes that were not directly presented by DEX_P&A2&N_. Consistently, interferon-γ (IFN-γ)-expressing CD8^+^ effector T cells and the level of IFN-γ were significantly elevated in blood from DEX_P&A2&N_-treated mice compared to DEX_P&A2_ and PBS groups (Additional file 1: Figure S3a and S3b). To translate this effect into a model closely mimicking clinical settings, we adopted an orthotopic HCC mouse model bearing large established tumors with tumor load of 0.64 ± 0.057 cm in longitudinal diameter formed in 21 days [11]. Remarkably, sustainable tumor suppression was observed in mice bearing large established tumors with DEX_P&A2&N_ (Fig. 3g–i), with greater than 71.4% of survival rate yielded in mice treated with DEX_P&A2&N_ on day 60, whereas no mice survived in DEX_P&A2_ and PBS groups (Fig. 3j), highlighting the potent antitumor immunogenicity of DEX_P&A2&N_ in orthotopic HCC mice.

### ***DEX***_***P&A&N***_*** eradicate tumor in orthotopic HCC mice***

**Fig. 4:**
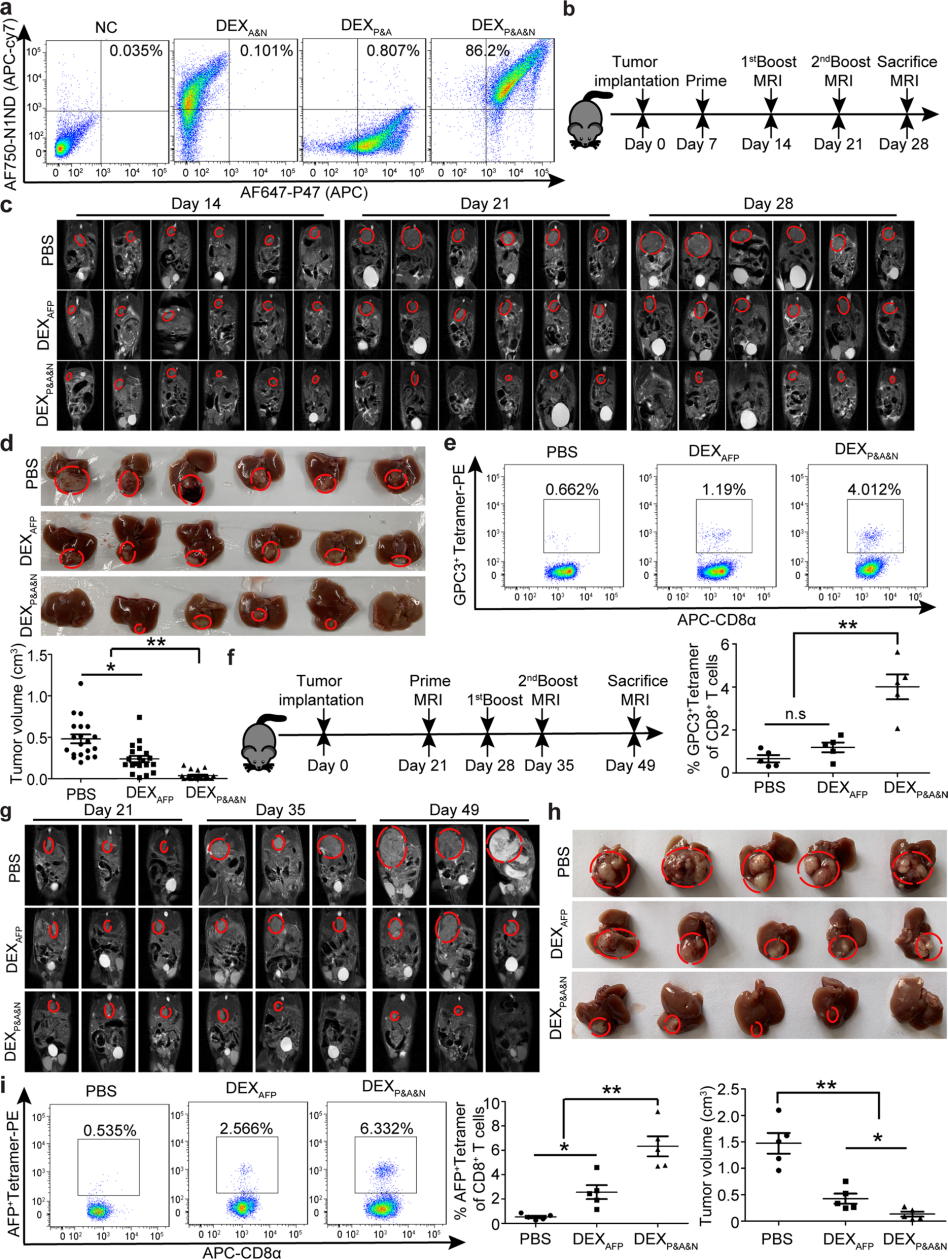
Antitumor immunity of DEX_P&A&N_ in orthotopic HCC mice. **a** Flow cytometric analysis to assess the double loading efficiency of two moieties on DEX_AFP_. DEX_P&A&N_ refer to DEX_P47&AFP&N1ND_. **b** Diagram for dosing regimen of DEX_P&A&N_. DEX_AFP_ or DEX_P&A&N_ (80 μg/mouse) were injected into day-7 orthotopic HCC mice intravenously three times weekly, and tissues were harvested 7 days after last injection. **c** Dynamic monitoring of tumor growth in orthotopic HCC mice with MRI at different time points. **d** Measurement of tumor size in orthotopic HCC mice at 21 days after priming (*n* = 20; one-way ANOVA on ranks). **e** Flow cytometric and quantitative analysis of GPC3^+^ tetramer T cells from tumor of orthotopic HCC mice treated with DEX_AFP_ or DEX_P&A&N_ (*n* = 5; one-way ANOVA post hoc Student–Newman–Keuls test). **f** Diagram for dosing regimen of DEX_P&A&N_. DEX_AFP_ or DEX_P&A&N_ (120 μg/mouse) were injected into day-21 orthotopic HCC mice bearing large established tumors intravenously three times weekly, and tissues were harvested 2 weeks after last injection. **g** Dynamic monitoring of tumor growth in orthotopic HCC mice bearing large established tumors treated with DEX_AFP_ or DEX_P&A&N_ with MRI at different time points. **h** Measurement of tumor size in orthotopic HCC mice at 28 days after priming (*n* = 5; one-way ANOVA post hoc Student–Newman–Keuls test). **i** Flow cytometric and quantitative analysis of AFP^+^ tetramer T cells from tumor of orthotopic HCC mice treated with DEX_AFP_ or DEX_P&A&N_ (*n* = 5; one-way ANOVA post hoc Student–Newman–Keuls test). *p < 0.05, **p < 0.001; n.s, not significant

Given the striking antitumor effect of DEX_P&A2&N_, we wondered whether replacing antigenic AFP epitope with the full-length AFP [16] will further enhance the immunogenicity of designer DEX vaccine. To validate this, we painted P47 and N1ND via CP05 on AFP-expressing DEX (DEX_AFP_) (Additional file 1: Figure S4a) to form DEX_P47&AFP&N1ND_ (DEX_P&A&N_), with a double loading efficiency of 86.2% (Fig. 4a). DEX_P&A&N_ showed tumor-targeting ability in day-14 orthotopic HCC mice bearing mCherry-expressing tumors (Additional file 1: Figure S4b and S4c), with a similar bio-distribution to DEX_P&A2&N_. Concordantly, DEX_P&A&N_ triggered persistent antitumor effect on day-7 orthotopic HCC mice, demonstrated by significant tumor retardation at different time points, with 20% of tumor-bearing mice becoming tumor-free on day 28 (Fig. 4b–d). Analysis of antigen-specific de novo T cell responses revealed significantly elevated numbers of CD8^+^ T cells specific for GPC3 epitope [31] in tumor-infiltrating lymphocytes from DEX_P&A&N_-treated mice than mice treated with DEX_AFP_ and PBS (Fig. 4e). Importantly, complete tumor eradication was observed in mice bearing large established tumors with tumor load of 0.62 ± 0.05 cm in longitudinal diameter after three repeated intravenous injections of DEX_P&A&N_ (Fig. 4f–h), with greater T cell responses specific for AFP epitope (Fig. 4i). These findings indicated that DEX_P&A&N_ enable the generation of antigen-specific antitumor immunity, resulting in tumor eradication in orthotopic HCC mice.

### ***DEX***_***P&A&N***_*** engage adaptive and innate immune responses***

**Fig. 5:**
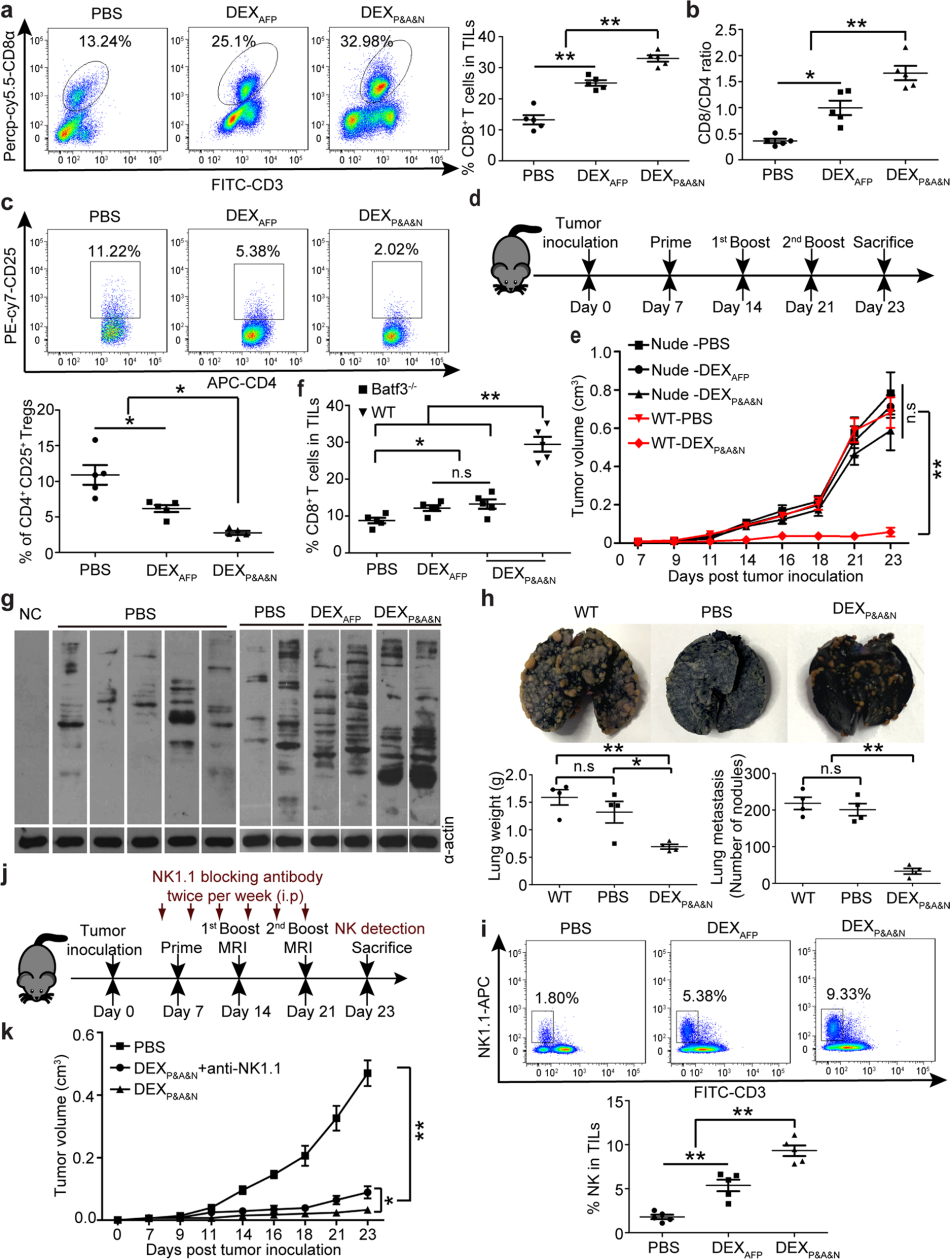
DEX_P&A&N_ evoke potent antitumor immunity with efficacy dependent on innate and adaptive immune responses. Flow cytometric and quantitative analysis of tumor-infiltrating CD8^+^ (**a**) and ratio of tumor-infiltrating CD8^+^ to CD4^+^ (**b**) and flow cytometric and quantitative analysis of tumor CD4^+^CD25^+^ (**c**) T cells of DEX_P&A&N_ -treated orthotopic HCC mice bearing large established tumors (*n* = 5). **d ** Diagram for dosing regimen of DEX_P&A&N_. DEX_AFP_ or DEX_P&A&N_ (80 μg) were injected into day-7 thymus-deficient nude mice bearing subcutaneous HCC tumors intravenously three times weekly, and tissues were harvested 2 days after last injection. **e** Measurement of tumor size in nude mice bearing subcutaneous HCC tumors at different time points (*n* = 5). One-way ANOVA on ranks test was used for day 7 and 21; one-way ANOVA post hoc Student–Newman–Keuls test was used for day 9, 11, 14, 16, 18 and 23. **f** Flow cytometric and quantitative analysis of tumor-infiltrating CD8^+^ T cells of DEX_P&A&N_-treated orthotopic Batf3^−/−^ HCC mice (*n* = 5; one-way ANOVA on ranks). **g** Western blot to examine Hepa1-6 tumor cell lysates with serum from DEX_P&A&N_- or DEX_AFP_-treated mice. α-actin was used as a loading control. **h** Representative images and quantification of tumor nodules in lungs 21 days after serum re-infusion and tumor challenge (*n* = 4). **i** Flow cytometric and quantitative analysis of tumor-infiltrating NK cells of DEX_P&A&N_-treated orthotopic HCC mice bearing large established tumors (*n* = 5). **j** Diagram for NK depletion experiments. **k** Measurement of tumor volume in DEX_P&A&N_ and anti-NK1.1-treated ectopic HCC mice at different time points (*n* = 5). One-way ANOVA post hoc Student–Newman–Keuls test was used for day 7, 9, 11, 14, 21 and 23; one-way ANOVA on ranks test was applied for day 16 and 18; and Two-tailed t test was applied for DEX_P&A&N_ with or without anti-NK1.1 antibody on day 23. *p < 0.05, **p < 0.001; n.s means not significant. Note: One-way ANOVA post hoc Student–Newman–Keuls test was used for Fig. 5a-c and 5 h, 5i

Analysis of tumor-infiltrating lymphocytes revealed a significant rise of CD8^+^ T cells and an increased ratio of CD8^+^ to CD4^+^ T cells (Fig. 5a, b), with drastic decline of CD25^+^CD4^+^ regulatory T cells (Tregs) (Fig. 5c) in DEX_P&A&N_-treated orthotopic HCC mice bearing large established tumors compared to DEX_AFP_ and PBS controls. Accordingly, levels of the immunostimulatory cytokine IFN-γ [32] significantly rose (Additional file 1: Figure S5a) and immunosuppressive cytokines transforming growth factor β (TGF-β) and interleukin-10 (IL-10) [33, 34] significantly declined (Additional file 1: Figure S5b) in tumor, suggesting that DEX_P&A&N_ remodel tumor microenvironment. To determine whether T cell-dependent adaptive immune responses are critical for the antitumor immunity, we intravenously administered DEX_P&A&N_ to day-7 thymus-deficient nude mice bearing subcutaneous HCC tumors (Fig. 5d). Remarkably, DEX_P&A&N_-mediated antitumor effect was largely abrogated in nude mice compared to wild-type mice, with no difference between DEX_P&A&N_ and DEX_AFP_ or PBS groups (Fig. 5e). Commensurately, dramatically reduced numbers of CD103^+^CD11c^+^ DCs in tumor from DEX_P&A&N_-treated Batf3^−/−^ mice (Additional file 1: Figure S5c and S5d) resulted in greatly decreased tumor-infiltrating CD8^+^ T cells (Fig. 5f) and tumor retardation (Additional file 1: Figure S5e), compared to wild-type mice, demonstrating that host Batf3-dependent cross-presenting DCs and T cells are primarily responsible for the antitumor immunity of DEX_P&A&N_. Notably, greater antitumor immune responses were observed in DEX_P&A&N_- and DEX_AFP_-treated Batf3^−/−^ mice than PBS controls, whereas no difference was found between DEX_P&A&N_ and DEX_AFP_ (Additional file 1: Figure S5e), implying that N1ND predominantly contributes to the recruitment of CD103^+^CD11c^+^ DCs and the antitumor immunogenicity of DEX_AFP_ is largely independent of CD103^+^CD11c^+^ DCs. In parallel, we evaluated endogenous antibody responses and the immunoblot with sera from DEX_P&A&N_-treated mice against Hepa1-6 cell lysates revealed that these endogenous antibodies recognized numerous antigens compared to mice treated with DEX_AFP_ or PBS controls (Fig. 5g). Consistently, re-infusion of sera from DEX_P&A&N_-immunized mice to naïve mice resisted tumor challenge with Hepa1-6 cells, reflected by significantly reduced lung weight and numbers of tumor nodules in lungs of mice treated with sera from DEX_P&A&N_-immunized mice compared to mice treated with sera from PBS controls or naive mice (Fig. 5h), suggesting generation of antibody responses to epitopes that were not directly targeted by DEX_P&A&N_. Analysis of natural killer (NK) cells, innate lymphoid cells bearing cytolytic capacity [35, 36], exhibited a significant increase in cell numbers in tumor from DEX_P&A&N_-treated mice bearing large established tumors compared to DEX_AFP_ and PBS controls (Fig. 5i) and depletion of NK cells [26, 37] (Additional file 1: Figure S5f) compromised the antitumor effect triggered by DEX_P&A&N_ on day 23 after tumor inoculation (Fig. 5j, k), suggesting that NK cells partially participated in DEX_P&A&N_-mediated antitumor immune responses. Altogether, these data showed that DEX_P&A&N_ can engage both adaptive and innate immune responses.

### Combination therapy resists tumor re-challenge and elicits long-lasting antitumor immunity in orthotopic HCC mice

**Fig. 6:**
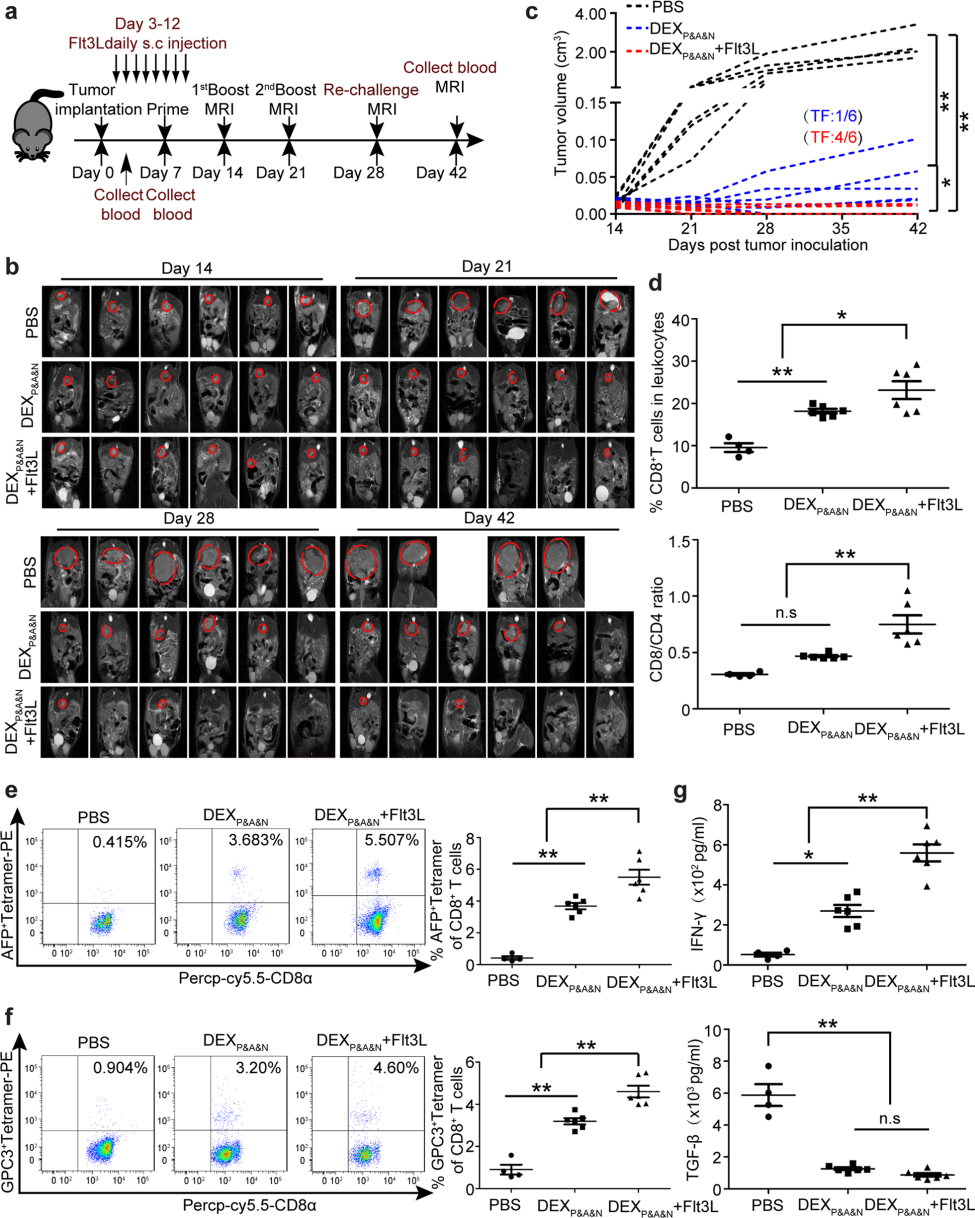
Flt3L augments DEX_P&A&N_’s antitumor potency in orthotopic HCC mice. **a** Diagram for dosing regimen of DEX_P&A&N_ and Flt3L in orthotopic HCC mice. Flt3L was administered subcutaneously to day-3 orthotopic HCC mice at the dose of 800 μg /kg/day for 8 days consecutively and DEX_P&A&N_ (80 μg/mouse) were intravenously administered on day 7 after tumor implantation three times weekly. **b** Dynamic monitoring of tumor growth in orthotopic HCC mice treated with DEX_P&A&N_ or DEX_P&A&N_ and Flt3L with MRI at different time points. **c** Measurement of tumor volume for each individual mouse treated with PBS, DEX_P&A&N_ or DEX_P&A&N_ and Flt3L (*n* = 6). One-way ANOVA on ranks test was used for day 21, 28 and 42; one-way ANOVA post hoc Student–Newman–Keuls test was used for day 14. TF means tumor-free. **d** Quantitative analysis of CD8^+^ T cells and ratio of CD8^+^ to CD4^+^ T cells in blood of orthotopic HCC mice treated with PBS (*n* = 4), DEX_P&A&N_ or DEX_P&A&N_ and Flt3L (*n* = 6) at day 42 after tumor implantation (One-way ANOVA on ranks). Flow cytometric and quantitative analysis of AFP^+^ tetramer (**e**) and GPC3^+^ tetramer (**f**) T cells in blood of orthotopic HCC mice treated with PBS (*n* = 4), DEX_P&A&N_ or DEX_P&A&N_ and Flt3L (*n* = 6) at day 42 after tumor implantation (one-way ANOVA post hoc Student–Newman–Keuls test). **g** Assessment of IFN-γ and TGF-β in blood of orthotopic HCC mice treated with PBS (*n* = 4), DEX_P&A&N_ or DEX_P&A&N_ and Flt3L (*n* = 6) at day 42 after tumor implantation (one-way ANOVA post hoc Student–Newman–Keuls test). *p < 0.05, **p < 0.001; n.s, not significant

Given that FMS-like tyrosine kinase 3 ligand (Flt3L) is an important growth factor to promote hematopoietic progenitor commitment to the DC lineage as well as DC survival and proliferation in tissues [38], we hypothesized that increasing the abundance of CD103^+^CD11c^+^ DCs will likely augment DEX_P&A&N_-mediated antitumor immunity. To test this, we subcutaneously injected Flt3L at the dose of 800 µg/kg/day for 8 days in day-3 orthotopic HCC mice [26, 39, 40] and primed mice 7 days after tumor implantation with intravenous injection of DEX_P&A&N_ (Fig. 6a). As expected, CD11c^+^ DCs including CD103^+^, CD8α^+^ and CD11b^+^ DCs were significantly expanded in blood of tumor-bearing mice after repeated subcutaneous injections of Flt3L (Additional file 1: Figure S6a-c), confirming the effectiveness of Flt3L. Strikingly, two-sixth of mice became tumor-free on day 21 after tumor implantation and the rate increased to four-sixth by day 28 (Fig. 6b, c). In addition, 66.7% of mice treated with DEX_P&A&N_ in combination with Flt3L were cured of primary tumors on day 28 and remained tumor-free 14 days after tumor re-challenge (Fig. 6b, c). Dynamic monitoring of immune microenvironment revealed significantly elevated numbers of CD8^+^ T cells and an increased ratio of CD8^+^ to CD4^+^ T cells (Fig. 6d), particularly antigen-specific tumor-reactive T cells (Fig. 6e, f), in which the level of circulatory IFN-γ significantly rose and TGF-β declined (Fig. 6g) in mice treated with DEX_P&A&N_ in combination with Flt3L compared to PBS controls on day 42, indicating a remodeling of immune microenvironment.

**Fig. 7:**
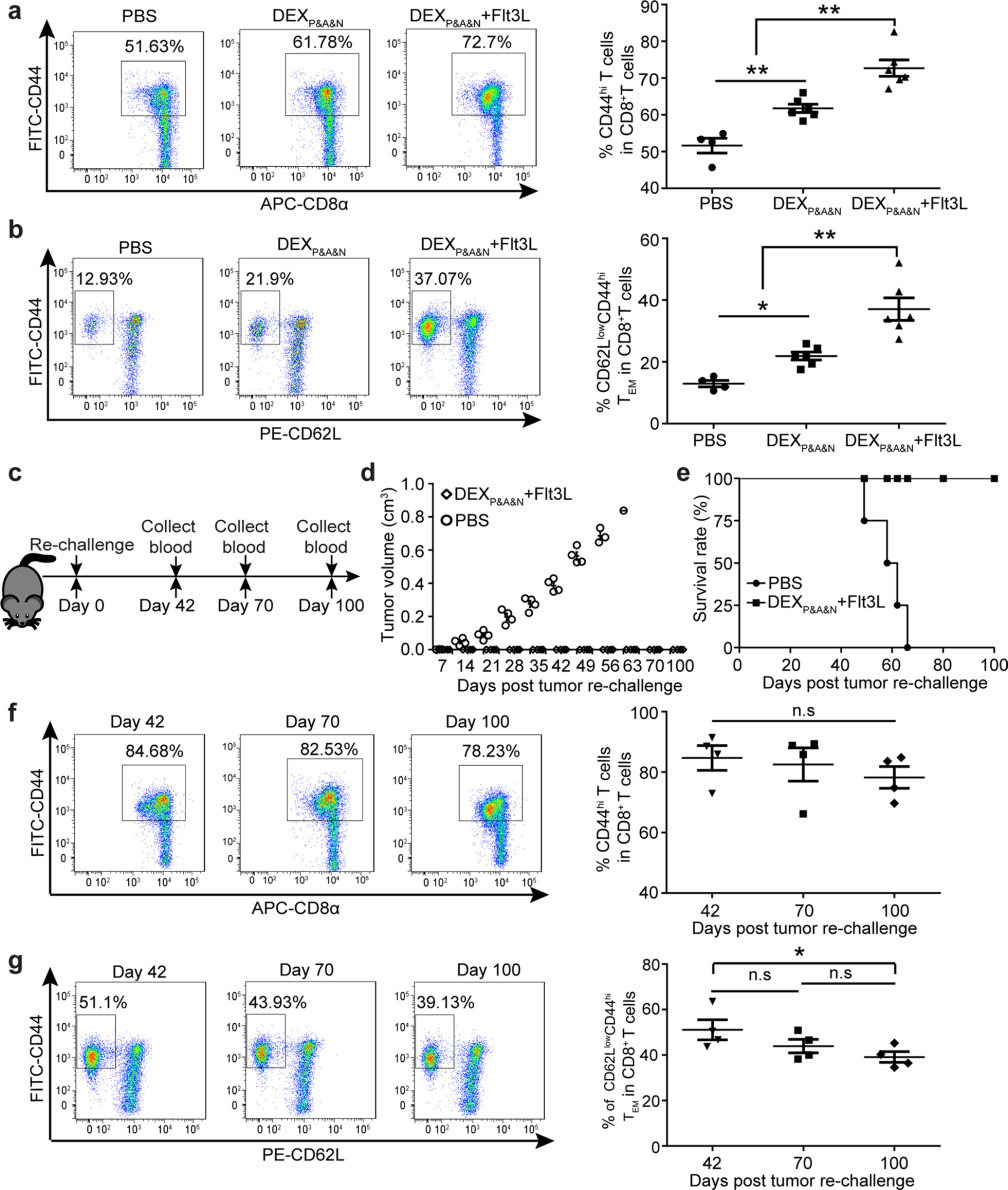
Long-term protective memory immune responses elicited by DEX_P&A&N_ and Flt3L against tumor re-challenge. Flow cytometric and quantitative analysis of CD44^hi^ CD8^+^ (**a**) and CD62L^low^CD44^hi^CD8^+^ (**b**) T cells in blood of orthotopic HCC mice treated with PBS (*n* = 4), DEX_P&A&N_, DEX_P&A&N_ and Flt3L (*n* = 6) at day 42 after tumor implantation (one-way ANOVA post hoc Student–Newman–Keuls test). **c** Diagram for tumor re-challenge in DEX_P&A&N_ and Flt3L-treated tumor-free mice. **d** Measurement of tumor volume in DEX_P&A&N_ and Flt3L-treated tumor-free mice after re-challenge (*n* = 4). Mann–Whitney rank-sum test was used for day 7; one-way ANOVA on ranks test was used for day 14, 21, 28, 35 and 42; one-way ANOVA post hoc Student–Newman–Keuls test was used for day 49. **e** Survival rate of DEX_P&A&N_ and Flt3L-treated tumor-free mice after re-challenge (*n* = 4). Flow cytometric and quantitative analysis of CD44^hi^CD8^+^ (**f**) and CD62L^low^CD44^hi^ CD8^+^ (**g**) T cells in blood of DEX_P&A&N_ and Flt3L-treated tumor-free mice at different time points after tumor re-challenge (*n* = 4; one-way ANOVA post hoc Student–Newman–Keuls test). *p < 0.05, **p < 0.001; n.s, not significant

To determine whether combinatorial treatment of DEX_P&A&N_ with Flt3L engendered long-term host memory antitumor responses, we analyzed memory T cells [41] in blood of mice treated with DEX_P&A&N_ and Flt3L at 35 days after initial priming. The results revealed significantly greater numbers of circulatory CD44^hi^CD8^+^ memory T cells (Fig. 7a), particularly CD62L^low^CD44^hi^ effector memory T cells (T_EM_) [41, 42] (Fig. 7b) in mice treated with DEX_P&A&N_ and Flt3L compared to DEX_P&A&N_ and PBS controls. In concert with generation of memory immune responses, we re-challenged the tumor-free survivors with 2 × 10^5^ Hepa1-6 cells subcutaneously on day 28 after initial tumor implantation (Fig. 7c). Strikingly, 100% of mice treated with DEX_P&A&N_ and Flt3L rejected tumor re-challenge and remained tumor-free at 100 days after cessation of treatment, whereas no mice survived in PBS groups on day 66 after tumor re-challenge (Fig. 7d, e), confirming the formation of effective immunological memory. Importantly, dynamic monitoring of circulating CD44^hi^CD8^+^ memory T cells and T_EM_ in mice treated with DEX_P&A&N_ and Flt3L at different time points exhibited stabilized pool of memory T cells, with a decline of T_EM_ on day 100 after re-challenge (Fig. 7f, g) [42], supporting the conclusion that sustainable immunological memory responses curtailed tumor re-challenge. Taken together, these data demonstrate that Flt3L enhances DEX_P&A&N_’s antitumor immunogenicity, resulting in long-lasting protective immunity.

## Discussion

**Fig. 8:**
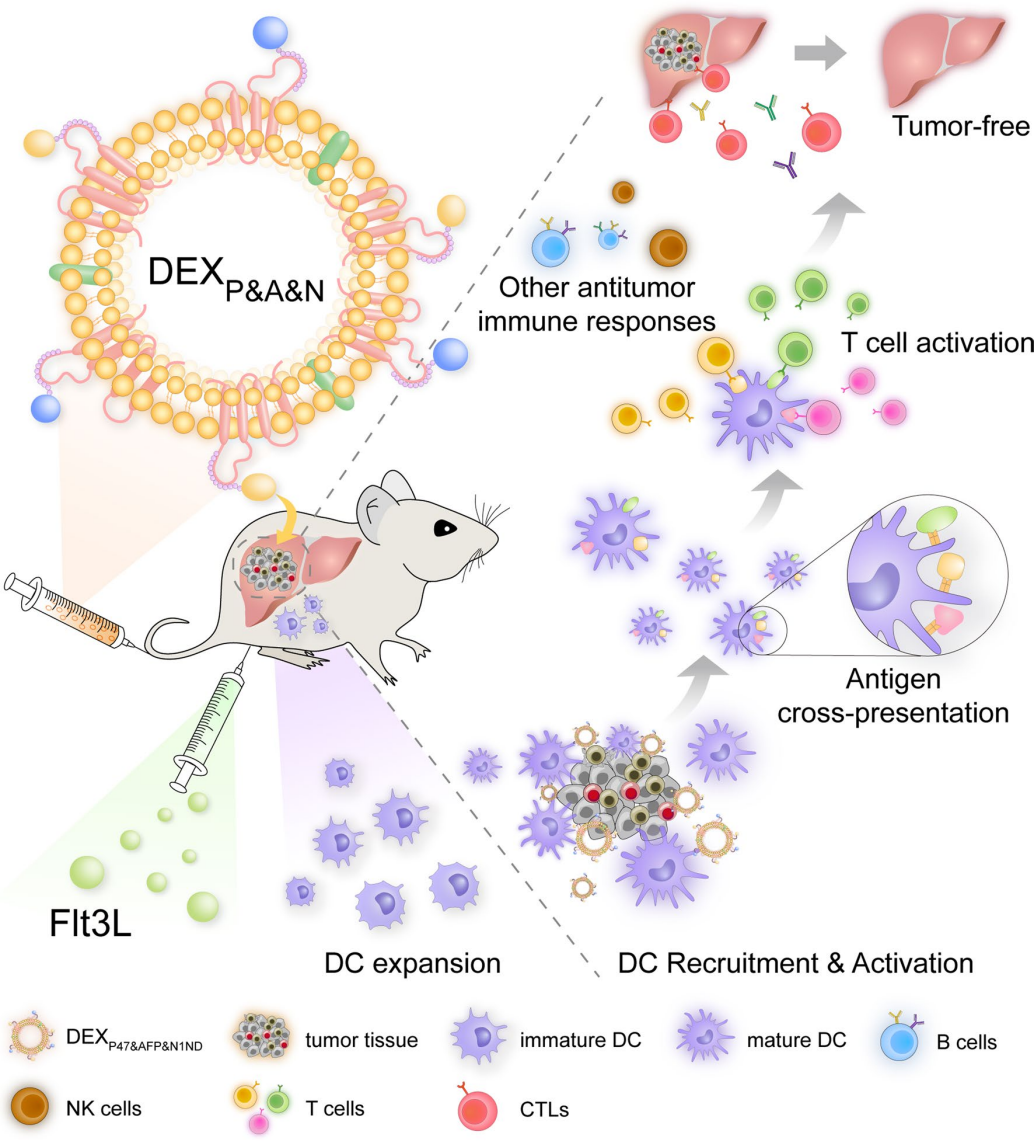
Graphical abstract for illustrating the mechanism underlying the antitumor immunogenicity of universal DEX vaccines

Although neoantigen-based personalized immunotherapy has attracted much attention, with a few ongoing clinical trials [3], antigen identification and tumor heterogeneity present a hurdle to designed antigen-based immunotherapy. An emerging concept is to actively stimulate the endogenous immune system to mount an effective antitumor response without employing specific antigens [39]. Here, we report that a universal DEX vaccine enables tumor-targeted endogenous DC recruitment, activation and cross-presentation of tumor antigens, and drives robust antitumor responses and even tumor eradication in HCC mice bearing large established tumors by engaging adaptive and innate immunity (Fig. 8). This is the first proof-of-principle study to demonstrate that it is feasible to achieve personalized immunotherapy without the need to identify tumor antigens. It represents a promising immunotherapeutic strategy to target genetically heterogeneous tumors and engage host antitumor immune responses beyond antigens encoded by the vaccine, thereby potentially reducing the occurrence of tumor escape. Importantly, this generalizable approach can be easily adapted to other tumors by replacing the tumor-targeting moiety and thus hold translational potential for tumors with high heterogeneity.

DEX, as promising acellular vaccines, have been tested in melanoma and non-small cell lung cancer patients with promising clinical outcomes, although the immunogenicity can be further enhanced [9, 43]. Consistent with previous clinical trials, our designer DEX vaccines also engaged NK cell- and antibody-mediated innate immunity, but T cell-dependent immune responses were largely responsible for the antitumor effect. Compared to DEX_AFP_ [16], DEX_P&A&N_ primarily depended on N1ND-mediated recruitment and activation of endogenous cross-presenting CD103^+^CD11c^+^ DCs, though other DC subsets such as CD11b^+^CD11c^+^ DCs were also involved. As CD11b^+^CD11c^+^ DCs were shown to mostly engage CD4^+^ effector T cell responses, whereas CD103^+^CD11c^+^ DCs primarily promoted engagement of host CD8^+^ T cell immunity [15], we focused on CD103^+^CD11c^+^ DCs in our study. Correspondingly, using Batf3^−/−^ strains as recipients, we confirmed that the functionality of DEX_P&A&N_ relied upon host CD103^+^CD11c^+^ DCs. Supplementation of Flt3L significantly amplified CD103^+^CD11c^+^ DCs and led to higher percentages of tumor-free rates in HCC mice, further underlining the importance of functional host Batf3-dependent DCs in driving DEX-mediated antitumor responses. Given that DCs are largely responsible for the functionality of this designer DEX vaccine, we combined clinically tested DC-booster Flt3L with DEX_P&A&N_ in the present study [44, 45]. However, as immune checkpoint inhibitors including programmed cell death 1 (PD-1) antibody have shown encouraging results in HCC patients when in combination with other therapeutic agents [46, 47], the combination therapy of DEX_P&A&N_ with anti-PD-1 antibody is warranted in future studies.

Notably, complete tumor eradication was achieved in DEX_P&A&N_-treated orthotopic HCC mice bearing large established tumors, which has not been possible with current immunotherapeutic approaches [48], highlighting the tenacity of HCC. Although a less pronounced antitumor effect was elicited by DEX_P&A2&N_, it is likely due to the comparatively low triple-loading efficiency of P47, AFP212 and N1ND on DEX. Although simultaneous loading of AFP212 and P47 on DEX was efficient at different ratios, the co-loading efficiency of P47 or AFP212 with N1ND was much lower (data not shown). N1ND’s larger molecular mass could potentially interfere with the loading of other moieties, although the loading of N1ND alone on exosomes was efficient [11]. Further optimization of loading ratios is warranted in future studies. Due to the small size of DEX, it remains challenging to quantify the distribution of each moiety in the exosome population. Nevertheless, DEX_P&A2&N_-mediated active recruitment and activation of endogenous DCs in tumor enhanced the overall efficacy of DEX vaccines in solid tumors such as HCC.

In summary, our study demonstrates a universal DEX vaccine bearing a targeting ligand, an antigen and a peptide immunoadjuvant that can trigger tailored response to each patient’s genetically heterogeneous tumor, engage host innate and adaptive immunity, and induce potent antitumor immune responses, resulting in tumor eradication in orthotopic HCC mice carrying large established tumors, and provide long-term protective immune memory against tumor re-challenge. Our study provides a generalizable approach for personalized tumor immunotherapy, particularly for tumors with high heterogeneity, paving the way for personalized tumor immunotherapy with a universal tumor vaccine, overcoming the regulatory hurdles to developing individualized diverse “personalized” vaccines. This work represents a leap in understanding for treatment of currently intractable tumors and may also pave the way for continual “boosting” of tumor immunotherapy without the need to fully characterize the tumor mutational profile or neoantigens. Moreover, this approach is generalizable to other tumors beyond HCC and can have a significant impact on the societal cancer burden.

## Methods

### Animals and injections

*C57BL/6* wild-type and thymus-deficient *BALB/C* nude mice (6–8 weeks old, purchased from Beijing Vital River Laboratory Animal Technology Co., Ltd, China) and *C57BL/6-Batf3*^em1Smoc^ (referred to Batf3^−/−^) (purchased from Shanghai Model Organisms Center Inc., China) were used in all experiments. (Number of animals used in each group was specified in corresponding figure legends.) All animal experiments were carried out in the animal unit of Tianjin Medical University (Tianjin, China) according to procedures authorized and specifically approved by the institutional ethical committee (permit SYXK2019-0004). For intravenous injections, various amounts of DEX_AFP_, DEX_A2&N_, DEX_P&A2_, DEX_P&A2&N_ or DEX_P&A&N_ in 200 μl phosphate-buffered saline (PBS) solutions were injected into tail vein of different tumor-bearing mice. Mice were killed by terminal anesthesia with isoflurane or 4% chloral hydrate. Tissues were harvested for isolation of living cells or fixed with Bouin’s solution (Sigma, USA) and embedded with paraffin for histological studies. Mouse recombinant Flt3/Flk-2 Ligand (STEMCELL Technologies Inc., Canada) was dissolved in saline for subcutaneous injection in day-3 tumor-bearing mice at the dose of 800 μg/kg/day for 8 days.

### Cell lines

The murine cell line DC2.4 (referred to as “DCs,” kindly provided by Dr. De Yang, Center for Cancer Research, National Institutes of Health, Bethesda, MD, USA) was used. DCs were cultured as described previously [49]. Briefly, DCs were cultured in Dulbecco’s modified Eagle’s medium (DMEM) with 1% penicillin and streptomycin (PS), 1% glutamine, 50 μM β-mercaptoethanol, and 10% fetal bovine serum (FBS) obtained by centrifugation at 100,000 g for 1 h to remove possible FBS-containing exosomes. α-fetoprotein (AFP)-expressing DCs (DC_AFP_) were generated and cultured as previously described [16]. The murine Hepa1-6 cell line (derived from *C57BL* mice, H-2K^b^) was purchased from Boster Biological Technology Ltd. (Wuhan, China). Human 293FT cell line used for lentivirus packaging was kept in house and cultured as described previously [16]. All cells except for DCs were grown at 37 °C in 5% CO_2_ in DMEM supplemented with 10% exosome-depleted FBS and 1% PS as described above. All the cell lines used were tested to rule out the presence of mycoplasm contamination.

### Preparation and purification of exosomes

Cell culture medium was sequentially centrifuged at 1000 g for 10 min, followed by 10,000 g for 30 min. The supernatant from different cells was collected and filtered with a 0.22-μm filter (Millex, Germany), followed by ultracentrifugation at 100,000 g for 1 h to pellet exosomes. Exosome pellets were washed in a large volume of PBS and recovered by centrifugation at 100,000 g for 1 h. Exosomes were further purified with sucrose gradient ultracentrifugation (Sigma, China) as previously described [14]. Exosome pellets were dissolved in PBS, and the total protein concentration of exosomes was quantified by the Bradford assay (Sangon Biotech, USA) and stored at − 80 °C.

### Characterization of exosomes

Exosome morphology was visualized using a high-resolution transmission electron microscope (TEM, Hitachi HT7700, Tokyo, Japan) as described previously [14]. Briefly, exosomes were resuspended and diluted into PBS (1 μg/μl) and mixed with an equal volume of 4% paraformaldehyde (PFA), followed by adsorption onto a glow-discharged, carbon-coated formvar film attached to a metal specimen grid. Excess solution was blotted off, and the grid was immersed with a small drop (50 μl) of 1% glutaraldehyde for 5 min, followed by washing with 100 μl distilled water eight times (2 min each time). Subsequently, the grid was transferred to 50 μl uranyl oxalate solution (pH7.0) for 5 min and then 50 μl methyl cellulose–uranyl acetate (100 μl 4% uranyl acetate and 900 μl 2% methyl cellulose) for 10 min at 4 °C. The excess solution was blotted off, and the sample was dried and examined with TEM. The size of exosomes was measured by Nano Particle Tracking and Zeta potential distribution analyzer (ParticleMetrix (PMX), Germany).

### Peptide binding assay

N1ND-CP05 (MPKRKVSSAEGAAKEEPKRRSARLSAKPPAKVEAKPKKAAAK DKSSDKKVQTCRHSQMTVTSRL) [10, 14], AFP212-CP05 (GSMLNEHVMCRHSQM TVTSRL) [13] and P47-CP05 (SQDIRTWNGTRSCRHSQMTVTSRL) [12] were purchased from China Peptide Ltd. (Suzhou, China) with > 95% purity. To measure the peptide binding efficiency, AF750-labeled N1ND-CP05 (20 μg) (kindly labeled by Professor Qibing Zhou, Huazhong University of Science and Technology, Wuhan, China), FAM-labeled AFP212-CP05 (20 μg) and AF647-labeled P47-CP05 (20 μg) (purchased from China Peptide Ltd., Shanghai, China) were incubated with DEX (DC-derived exosomes) (10 μg) overnight at 4 °C, followed by washing with PBS for five times in 2-ml ultracentrifuge tubes and filtered with 100-kDa diafiltration tube (Millipore, USA) to remove unbound peptides. The same protocol was also applied to peptide loading on DEX_AFP_. Subsequently, the binding efficiency of peptide–exosome complexes was measured by flow cytometry (BD LSRFortessa™ cell analyzer, USA).

### Western blot

Exosome and cell pellets were lysated in lysis buffer (125 mM Tris–HCl, pH6.8, 10% sodium dodecyl sulfate (SDS), 2 M urea, 20% glycerol and 5% β-mercaptoethanol) and subjected to 10% SDS–polyacrylamide gel electrophoresis, and gels were transferred to a PVDF membrane. Membranes were blocked in 5% skimmed milk and probed with primary antibodies including mouse monoclonal antibodies for Alix (1:1000, Cell Signaling Technol., USA) and CD81 (1:200, Santa Cruze, USA); and rabbit polyclonal antibodies for CD63 (1:200, Santa Cruze, USA), AFP (1:1000, Abcam, USA) and Cytochrome C (1:1000, Cell Signaling Technol., USA) overnight at 4 °C. The bound primary antibody was detected by horseradish peroxidase (HRP)-conjugated goat anti-mouse, anti-rabbit or rabbit anti-mouse IgG (1:5000, Sigma, USA), respectively. The ECL western blotting analysis system (Millipore, Billerica, MA, USA) was applied.

### ***Generation of OVA***^+^***or mCherry***^+^***Hepa1-6 cell line***

Ovalbumin (OVA) plasmid was kindly provided by Professor Ting Wang (Tianjin Medical University, Tianjin, China). To obtain OVA-expressing lentiviruses, human 293FT cells (5 × 10^7^) were seeded in a 10-cm Petri dish for 24 h, followed by co-transfection with pCDH-CMV-GFP-OVA-puro, ΔR and VSVG plasmids in a mass ratio of 2:1:1 (µg) by calcium phosphate precipitation. Viruses were harvested and titred 48 h later and used for subsequent infection of Hepa1-6 cells. MCherry (pCDH-CMV-mCherry-puro)-expressing lentiviruses were purchased from HanBio Company (Shanghai, China). Hepa1-6 cells (1 × 10^5^) were seeded on 12-well plate and infected with 5 × 10^7^ (PFU) of OVA- or mCherry-expressing lentiviruses for 12 h. The infection was repeated three times every 24 h apart, followed by puromycin selection (2 µg/ml) for 2 weeks. mCherry^+^ or OVA-GFP^+^ cells were sorted and collected with a FACS Aria (BD Instruments, USA).

### Establishment of ectopic or orthotopic HCC mouse models

Ectopic mouse models were established by subcutaneous injection of 0.1 ml PBS containing Hepa1-6 cells (5 × 10^5^) into the left axilla of *C57BL/6* male mice with or without NK cell depletion or thymus-deficient *BALB/C* nude mice. When tumors reached 10–20 mm^3^, tumor-bearing mice were treated with PBS, DEX_AFP_, DEX_P&A2_, DEX_P&A2&N_ or DEX_P&A&N_ intravenously at the dose of 80 or 120 µg/mouse/week for 3 weeks, respectively. Mice were monitored during the treatment and killed on day 23 after inoculation. The length (L) and width (W) of tumors were measured with a caliper. Tumor size was calculated by the formula: (L × W^2^)/2 as previously described [50]. To establish orthotopic HCC mouse models, subcutaneous tumors with a longitudinal diameter of 1 cm were peeled from mice bearing Hepa1-6, mCherry^+^ Hepa1-6 and OVA^+^ Hepa1-6 tumor. Tumor tissues were washed in D-Hanks’ buffer, and necrotic tissues were removed from tumors. Subsequently, tumor tissues were cut into about 1 mm^3^ pieces, and two to three tumor pieces were implanted in the left lobe of the liver in recipient mice (*C57BL/6* or *C57BL/6-Batf3*^−/−^) under anesthesia.

### Magnetic resonance imaging (MRI) assessment

The magnetic resonance images of orthotopic HCC mice were acquired using T2 propeller sequence with the following parameters: slice thickness of 1.0 mm, slice spacing of 0.5 mm, TR/TE of 3494/70.7 ms, matrix of 256 × 160 and FOV of 8 × 8 cm (3.0 Tesla MR scanner, Signa Excite HDx; GE Healthcare, Milwaukee, WI, USA) with a small animal coil in Tianjin Medical University General Hospital as previously described [11]. During the examination, mice were anesthetized with pentobarbital sodium and fixed to minimize body motion, and respiration rate was monitored and body temperature was maintained to be at 37 °C using warm airflow.

### Tissue distribution

To examine the bio-distribution of DEX_P&A2&N_, DEX_A2&N_, DEX_AFP_ or DEX_P&A&N_ in orthotopic HCC mice bearing mCherry-expressing tumor. DiR-labeled DEX_P&A2&N_, DEX_A2&N_, DEX_AFP_ or DEX_P&A&N_ (80 μg) was administered into day-14 orthotopic HCC mice bearing mCherry^+^ tumor for single intravenous injection, respectively. Perfusion was performed 2 h after injection with 50 ml cold PBS to wash out DEX in circulation. Heart, muscle, liver, tumor, mesenteric lymph node, inguinal lymph node, kidney and spleen were harvested for imaging with IVIS spectrum (PE, Waltham, MA, USA).

### Isolation of lymphocytes in peripheral blood/lymphoid organs/tumor tissues

Peripheral blood from HCC mice treated with different vaccines was collected with 1% heparin. For isolation of mononuclear cells, collected peripheral blood was added gently on the top of equal volume of Lymphoprep™ (STEMCELL Technologies Inc., Canada) and centrifuged at 3000 g for 5 min, followed by lysis with the ammonium–chloride–potassium (ACK) lysis buffer for 5 min at room temperature (RT) to generate lymphocyte suspensions. For isolation of lymphocytes from spleen or draining lymph nodes, tissues were harvested and washed three times with PBS containing 2% FCS and 2% PS. Single lymphocyte cell suspensions were obtained by rubbing and pressing lymph nodes on 70-μm nylon sieve (BD Biosciences, Belgium) using a pair of sterile forceps and syringe handle. Subsequently, cells were re-suspended in RPMI 1640 medium containing 10% FCS, 2 mM L-glutamine, 50 μM β-mercaptoethanol and 1% PS. Mixture of lymphocytes was used for antibody staining and flow cytometry (BD, USA) detection as described below. Isolation of lymphocytes from tumor tissues was performed as previously described [11]. Briefly, tumor tissues were minced into small pieces with surgical scissors, followed by dissociation into single-cell suspensions with mechanical dissociation and enzymatic degradation of the extracellular matrix with Tumor Dissociation Kit (Miltenyi Biotec, Germany) as per manufacturer’s instructions. After dissociation, the sample is applied to 70-μm cell strainer to remove any remaining larger particles from the single-cell suspension. The extract was centrifuged at 528 g for 10 min, and the supernatant was removed. The mixture was re-suspended with 10 ml 40% Percoll (Pharmacia, Sweden), followed by centrifugation at 850 g for 30 min at 22 °C to remove the supernatant. Cell pellets were re-suspended in ACK lysis buffer to remove red blood cells, and the rest cells were used for antibody staining.

### *T cell division and antigen-specific reactivity assay *in vitro

T lymphocytes were harvested from tumor-draining lymph nodes or spleen of tumor-bearing mice treated with different vaccines 48 h after last injection. T lymphocytes (1 × 10^7^) were stained with 200 nM carboxyfluorescein diacetate succinimidyl ester (CFSE) (Life Technology, USA) as per manufacturer’s instructions. CFSE-labeled T cells (3 × 10^5^) were incubated with 10 μM GPC3 epitopes (40 μg/ml) for 72 h as reported previously [29]. Subsequently, APC-cy7-anti-mouse CD45 (1:1000, BioLegend, USA), PE-anti-mouse CD3 (1:500, eBioscience, USA), Percp-cy5.5 or APC-anti-mouse-CD8α (1:500, BioLegend, USA) and PE-cy7-anti-mouse-IFNγ (1:500, BioLegend, USA) antibodies were used to counter-stain antigen-specific T cells at 4 °C for 30 min, followed by flow cytometric (BD LSRFortessa™ cell analyzer, USA) analyses.

### Flow cytometry and intracellular staining

For the detection of mouse DC surface markers, DCs were washed in the FACS buffer (PBS containing 0.5% BSA and 0.05% NaN_3_), stained with a combination of FITC-, PE-cy7- or PE-anti-mouse IgG2b Isotype (1:500, eBioscience, USA), APC-anti-mouse IgG2b Isotype (1:500; BioLegend, USA), FITC- or APC-cy7-anti-mouse CD45 (1:1000, BioLegend, USA), PE-anti-mouse CD11c (1:500, BioLegend, USA), BV421- or PE-cy7- anti-mouse CD11b (1:1000, BioLegend, USA), APC- or Percp-cy5.5-anti-mouse CD8α (1:500, BioLegend, USA), APC-cy7-anti-mouse MHC-I (1:250, eBioscience, USA), APC-anti-mouse CD86 (1:250, eBioscience, USA), PE-cy7-anti-mouse CCR7 (1:250, BioLegend, USA), APC-anti-mouse CD103 (1:250; BioLegend, USA) on ice for 30 min prior to flow cytometry. For detection of subsets of leukocytes including NK cells and T lymphocytes in peripheral blood /lymphoid organs /tumor tissues, single-cell suspensions were stained with FITC- or APC-cy7-anti-mouse CD45 (1:500, BioLegend, USA), Percp-cy5.5- or FITC- or PE-anti-mouse CD3 (1:500, eBioscience, USA), APC-anti-mouse NK1.1 (1:500, eBioscience, USA), APC- or PE-anti-mouse CD4 (1:500, eBioscience, USA), PE-cy7-anti-mouse CD25 (1:500, eBioscience, USA), APC- or Percp-cy5.5-anti-mouse CD8α (1:500, BioLegend, USA), PE-anti-mouse CD62L (1:500, eBioscience, USA) or FITC-anti-mouse CD44 (1:500, eBioscience, USA) antibodies. Cells were washed with the FACS buffer three times after staining, followed by flow cytometry (BD LSRFortessa™ cell analyzer, USA). All data analyses were performed using FlowJo software (Tree Star Inc., Ashland, OR, USA). Intracellular staining of IFN-γ was performed as per manufacturer’s instructions (BD Biosciences, USA). Briefly, lymphocytes were collected from spleen, tumor tissues or peripheral blood of treated mice at different time points and transferred into U-bottom 96-well plates in 200 μl T cell culture medium (RPMI 1640 supplemented with 10% FBS, 1% PS and 0.05 mM β-mercaptoethanol). Subsequently, lymphocytes were pulsed with different antigen epitopes (40 μg/mL) for 16 h, followed by the addition of Ionomycin (400 ng/mL, Cayman, USA) for 4 h in the presence of GolgiPlug Protein Transport Inhibitor (1.5 μg/ml, GolgiStop, BD Biosciences, USA) for 6 h to block cytokine secretion. Cells were collected, washed and stained with FITC-anti-mouse CD3 (1:500, eBioscience, USA) and Percp-cy5.5-anti-mouse CD8α (1:500, BioLegend, USA) at 4 °C for 30 min. Subsequently, cells were washed and fixed with Cytofix (BD Biosciences, USA), followed by permeabilization with the permeabilization solution (BD Biosciences, USA) for another 20 min at 4 °C. Permeabilized cells were further stained with PE-cy7-anti-mouse IFN-γ antibody (1:500, eBioscience, USA) as per manufacturer’s instructions. Flow cytometry data were acquired by BD FACSVerse or LSRFortessa (BD, USA) and analyzed with FlowJo software (FlowJo, LLC).

### *Cytokine release assay *in vitro

Immune cytokines were detected by ELISA as described previously [11]. Briefly, mouse blood or tumor tissues were harvested from treated orthotopic HCC mice 48 h after last immunization. Blood was centrifuged at 3000 g for 30 min at RT, followed by analysis of IFN-γ, IL-10 and TGF-β with ELISA kits (eBioscience, USA) as per manufacturer’s instructions, respectively. Tumor tissues (150 mg) were homogenized with the homogenizer and then subjected to centrifugation at 3000 g for 30 min at 4 °C, followed by analysis of IFN-γ, IL-10 and TGF-β with ELISA kits as described above.

### Tetramer staining

Mouse CD8^+^ T cells were stained with antigen-specific tetramers as described previously [11]. Briefly, AFP-, GPC3- and OVA-specific tetramers were generated as per manufacturer’s instructions (the QuickSwitchTM Quant H-2K^b^ tetramer kit, TB-7400-K1, MBL, USA) with AFP212 (GSMLNEHVM) [13], GPC3 (AMFKNNYPSL) [31] and OVA (SIINFEKL) [28] (10 μM) epitopes, respectively. Lymphocytes derived from treated mice were treated with 50 nM dasatinib (HY-10181, MCE, USA) for 30 min at 37 °C, followed by washing with 1X washing buffer and stained with AFP-H-2K^b^-tetramer-PE, GPC3-H-2K^b^-tetramer-PE or OVA-H-2K^b^-tetramer-PE (2 μg/ml) for 60 min at 4 °C. Subsequently, tetramer-stained lymphocytes were counterstained with APC-cy7-CD45 (eBioscience, USA), FITC-anti-mouse CD3 (eBioscience, USA) and APC-anti-mouse CD8α (BioLegend, USA) antibodies. Stained cells were subjected to flow cytometric analysis with BD LSRFortessa (BD, USA) and analyzed by FlowJo software (FlowJo, LLC).

### Antibody depletion

To deplete NK cells, anti-mouse NK1.1 antibodies (BioXCell, USA) (400 μg/mouse twice per week for 3 weeks) were intraperitoneally administered into day-6 *C57BL/6* mice bearing ectopic HCC tumor, followed by intravenous immunization of PBS, DEX_AFP_ or DEX_P&A&N_ at the dose of 80 μg/mouse/week for 3 weeks one day later. NK cells were isolated from peripheral blood of tumor-bearing mice on day 23 after tumor inoculation and analysis by flow cytometry as described above.

### Endogenous antibody detection and serum transfer

Hepa1-6 cells were lysated in lysis buffer (125 mM Tris–HCl, pH6.8, 10% SDS, 2 M urea, 20% glycerol and 5% β–mercaptoethanol) and subjected to 10% SDS-polyacrylamide gel electrophoresis, and gels were transferred to a PVDF membrane. Membranes were blocked with 5% skimmed milk for 2 h at 4 °C and then stained with serum from DEX_AFP_- or DEX_P&A&N_-treated mice (day-49 tumor-bearing mice) and rabbit anti-α-actin antibody overnight at 4 °C. Membranes were washed and stained with goat anti-mouse IgG (1:5000, Sigma, USA) and goat anti-rabbit IgG (1:5000, Sigma, USA) for 2 h at 4 °C. The ECL western blot analysis system was used. For the serum transfer experiment, day-21 orthotopic HCC mice bearing large established tumors were treated with PBS or DEX_P&A&N_ at the dose of 120 μg/mouse/week for 3 weeks and serum was collected from day-49 treated orthotopic HCC mice or from age-matched wild-type *C57BL/6* mice, and complement was inactivated by incubation at 57 °C for 30 min. Serum (350 μl) was transferred into naive recipients (*C57BL/6* mice) intraperitoneally 6 h before intravenous injection of 5 × 10^5^ Hepa1-6 cells. Lungs were harvested 21 days after serum transfer, and lung nodules were counted in a double-blinded fashion after tracheal ink (1:10 diluted in PBS) injection and fixation with Fekete’s solution (5 ml 70% ethanol, 0.5 ml formalin and 0.25 ml glacial acetic acid)^51^.

### Statistical analysis

All data are reported as mean values ± SEM. Statistical differences between treated and control groups were evaluated by SigmaStat (Systat Software, London, UK). Both parametric and nonparametric analyses were applied as specified in figure legends. Sample size was determined by G*Power 3.1.7 (Power analysis and Sample size). Significance was determined based in *p* < 0.05.

## Supplementary Information

**Additonal file 1.** Supplementary Figures.

## Data Availability

Not applicable.

## Abbreviations

HCC: Hepatocellular carcinoma
DC: Dendritic cell
DEX: Dendritic cell (DC)-derived exosomes
P47-P: HCC-targeting peptide
AFP212-A2: α-Fetoprotein epitope
N1ND-N: High mobility group nucleosome-binding protein 1
AFP (A): Full-length AFP
DEX_AFP_: AFP-expressing DEX
IFN-γ: Interferon-γ
TGF-β: Transforming growth factor β
IL-10: Interleukin-10
Flt3L: FMS-like tyrosine kinase 3 ligand
