# Supplementary material for: Universal immunotherapeutic strategy for hepatocellular carcinoma with exosome vaccines that engage adaptive and innate immune responses
Source: J Hematol Oncol. 2022 Apr 29;15:46. doi: 10.1186/s13045-022-01266-8 (PMC9052531; doi:10.1186/s13045-022-01266-8)
Supplement: Supplementary file 1 — Additonal file 1. Supplementary Figures. [file 13045_2022_1266_MOESM1_ESM.pdf]

## Supplementary Figures

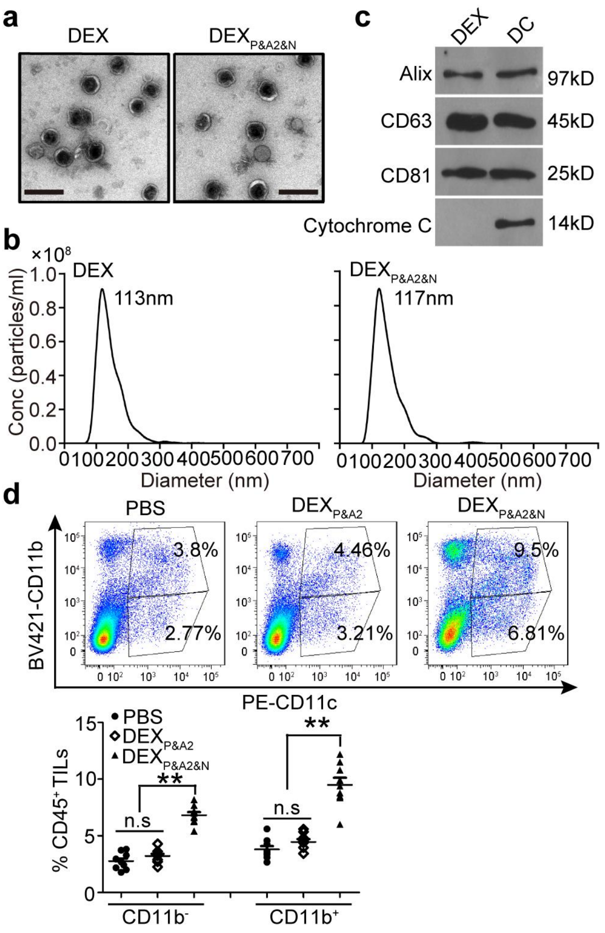


**Supplementary Figure 1**. Characterization and evaluation of DEX$_{P\&A2\&N}$ to recruit DCs to tumor of orthotopic mCherry-expressing HCC mice. (**a**) Representative transmission electron microscopic (TEM) images of DEX and DEX$_{P\&A2\&N}$ (scale bar= 200nm). P-P47; A2-AFP212; N-N1ND. DEX refers to DC-derived exosomes. (**b**) Size distribution of DEX and DEX$_{P\&A2\&N}$ with nanoparticle tracking analysis (NTA). (**c**) Western blot to examine the expression of exosomal biomarkers. Total protein (30 μg) was loaded for DEX and DC. (**d**) Flow cytometric and quantitative analysis of CD11b$^+$CD11c$^+$ and CD11b$^-$CD11c$^+$ DCs from tumor of orthotopic HCC mice treated with DEX$_{P\&A2}$ or DEX$_{P\&A2\&N}$ (n=9; One way-ANOVA post hoc Student-Newman -Keuls test). $^{**}p<0.001$; n.s means not significant.

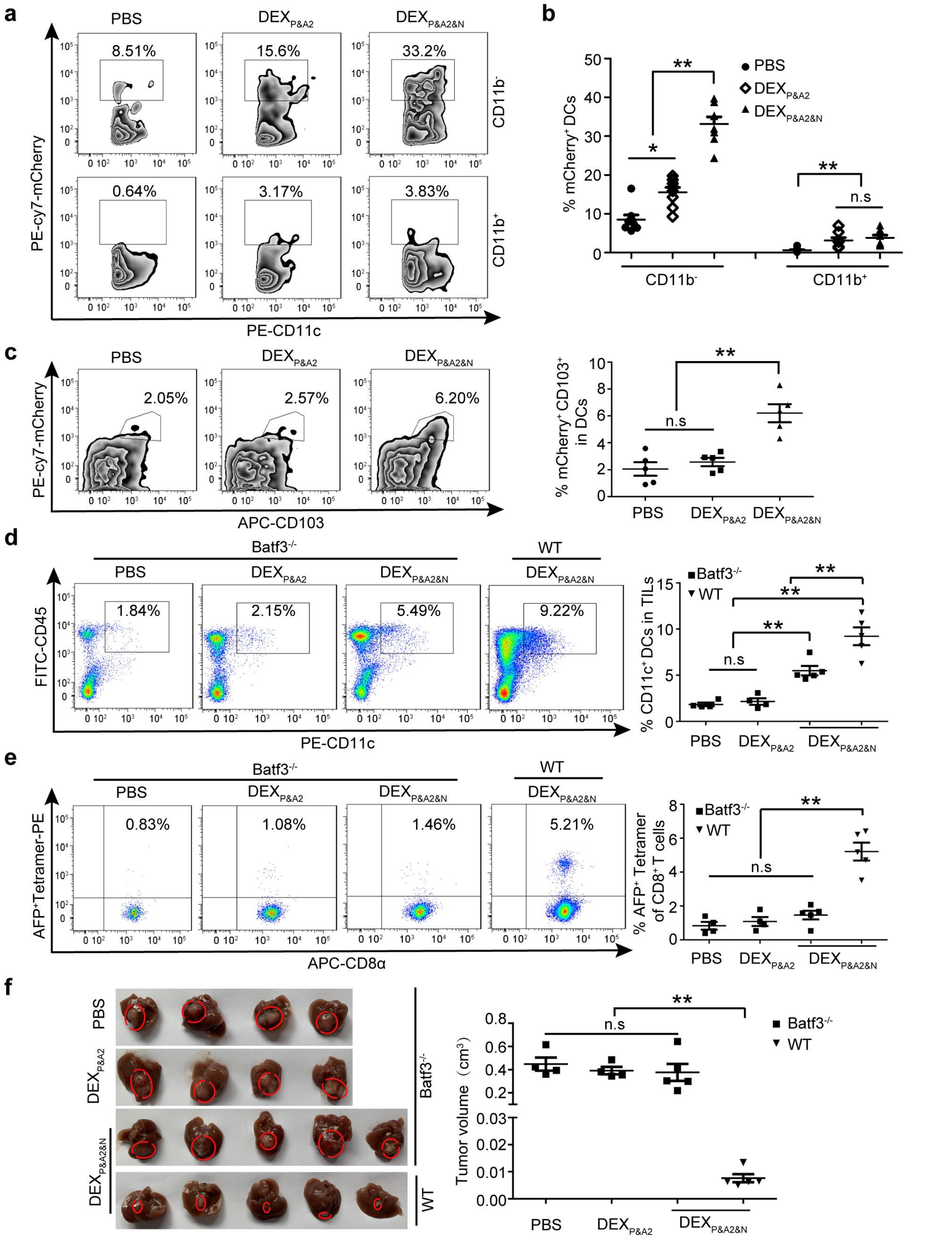


**Supplementary Figure 2**. Effects of $DEX_{P\&A2\&N}$-mediated DC cross-presentation and antitumor responses in orthotopic HCC mice. Flow cytometric (**a**) and quantitative analysis (**b**) of $mCherry^{+}CD11b^{+}CD11c^{+}$ or $mCherry^{+}CD11b^{-}CD11c^{+}$ DCs from tumor of orthotopic HCC mice treated with $DEX_{P\&A2}$ or $DEX_{P\&A2\&N}$ (n=8; One way-

ANOVA post hoc Student-Newman-Keuls test). (**c**) Flow cytometric and quantitative analysis of mCherry$^+$CD103$^+$CD11c$^+$ DCs in splenocytes of orthotopic HCC mice treated with DEX$_{P\&A2}$ or DEX$_{P\&A2\&N}$ (n=5; One way-ANOVA post hoc Student-Newman-Keuls test). (**d**) Flow cytometric and quantitative analysis of CD11c$^+$ DCs from tumor- infiltrating lymphocytes (TILs) of orthotopic Batf3$^{-/-}$ HCC mice treated with PBS (n=4), DEX$_{P\&A2}$ (n=4) or DEX$_{P\&A2\&N}$ (n=5) and orthotopic wild-type (WT) HCC mice treated with DEX$_{P\&A2\&N}$ (n=5) (One way-ANOVA post hoc Student-Newman-Keuls test). (**e**) Flow cytometric and quantitative analysis of AFP$^+$ tetramer T cells from tumor of orthotopic Batf3$^{-/-}$ HCC mice treated with PBS (n=4), DEX$_{P\&A2}$ (n=4) or DEX$_{P\&A2\&N}$ (n=5) and orthotopic WT HCC mice treated with DEX$_{P\&A2\&N}$ (n=5) (One way-ANOVA post hoc Student-Newman-Keuls test). (**f**) Representative tumor images and tumor volume of orthotopic Batf3$^{-/-}$ HCC mice treated with PBS (n=4) DEX$_{P\&A2}$ (n=4) or DEX$_{P\&A2\&N}$ (n=5) and orthotopic WT HCC mice treated with DEX$_{P\&A2\&N}$ (n=5) (One way-ANOVA post hoc Student-Newman-Keuls test). *$p<0.05$, **$p<0.001$; n.s means not significant.

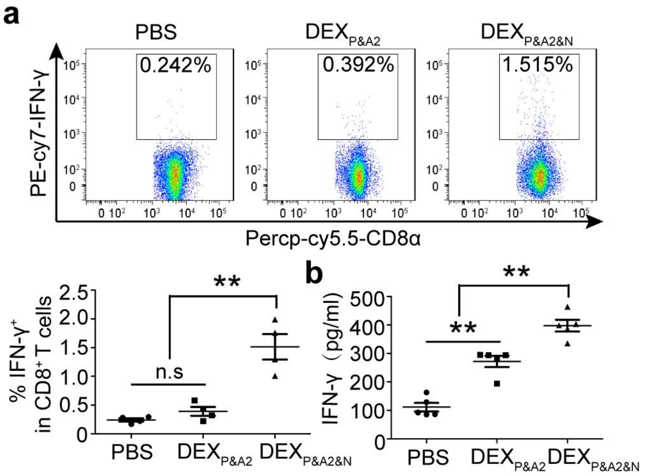


**Supplementary Figure 3**. Analysis of immune microenvironment of orthotopic HCC mice treated with $DEX_{P\&A2\&N}$. (**a**) Flow cytometric and quantitative analysis of IFN-$\gamma^{+}$CD8$^{+}$ T cells in blood of orthotopic HCC mice treated with $DEX_{P\&A2}$ or $DEX_{P\&A2\&N}$ (n=4; One way-ANOVA post hoc Student-Newman-Keuls test). (**b**) Measurement of IFN-γ in blood of orthotopic HCC mice treated with $DEX_{P\&A2}$ or $DEX_{P\&A2\&N}$ (n=5; One way-ANOVA post hoc Student-Newman-Keuls test). **$p<0.001$; n.s means not significant.

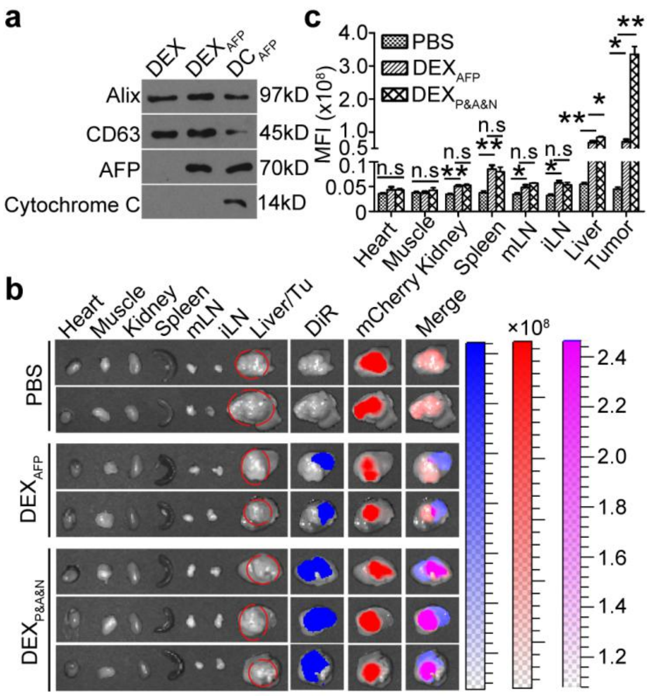


**Supplementary Figure 4**. Western blot analysis and tissue distribution of $DEX_{AFP}$ and $DEX_{P\&A\&N}$. (**a**) Western blot to examine the expression of AFP on DEX. Total protein (30 μg) was loaded for DEX and DC. DEX refer to DC-derived exosomes. Tissue distribution (**b**) and quantitative analysis (**c**) of labeled $DEX_{P\&A\&N}$. DiR-labeled $DEX_{AFP}$ or $DEX_{P\&A\&N}$ (80 μg/mouse) were injected into day-14 orthotopic HCC mice bearing mCherry-expressing tumors intravenously and tissues were harvested 2 h after injection (n=3; One way-ANOVA post hoc Student-Newman-Keuls test). mLN-mesenteric lymph node; iLN-inguinal lymph node. Tumor was circled. *p<0.05, **p<0.001; n.s means not significant.

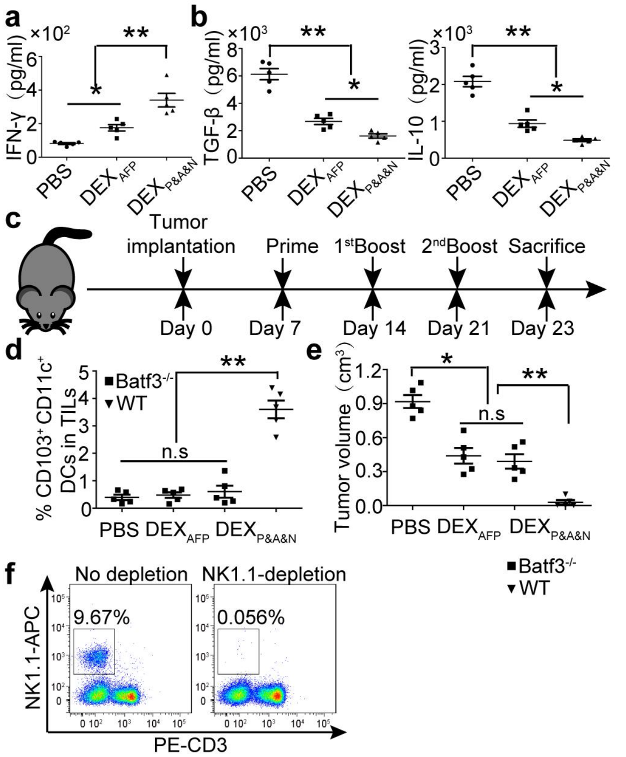

**Supplementary Figure 5**. Examination of tumor microenvironment in DEX$_{P\&A\&N}$-treated orthotopic HCC mice bearing large tumors and NK depletion. Measurement of IFN-γ (**a**) and TGF-β and IL-10 (**b**) in tumor of DEX$_{AFP}$- or DEX$_{P\&A\&N}$-treated orthotopic HCC mice bearing large established tumors (n=5). (**c**) Diagram for dosing regimen of DEX$_{P\&A\&N}$ in orthotopic Batf3$^{-/-}$ HCC mice. DEX$_{AFP}$ or DEX$_{P\&A\&N}$ (80 μg/ mouse) were intravenously injected into day-7 orthotopic Batf3$^{-/-}$ HCC mice weekly for 3 times and tissues were harvested 2 days after last injection. (**d**) Quantitative analysis of CD103$^{+}$CD11c$^{+}$ DCs from TILs of DEX$_{P\&A\&N}$-treated orthotopic Batf3$^{-/-}$ and wild-type (WT) HCC mice (n=5). (**e**) Measurement of tumor size in DEX$_{P\&A\&N}$-treated orthotopic Batf3$^{-/-}$ and WT HCC mice (n=5). (**f**) Flow cytometric analysis of NK cells in blood of orthotopic HCC mice on day 23 after tumor inoculation. *p<0.05, **p<0.001; n.s means not significant; One way-ANOVA post hoc Student-Newman-Keuls test was used.

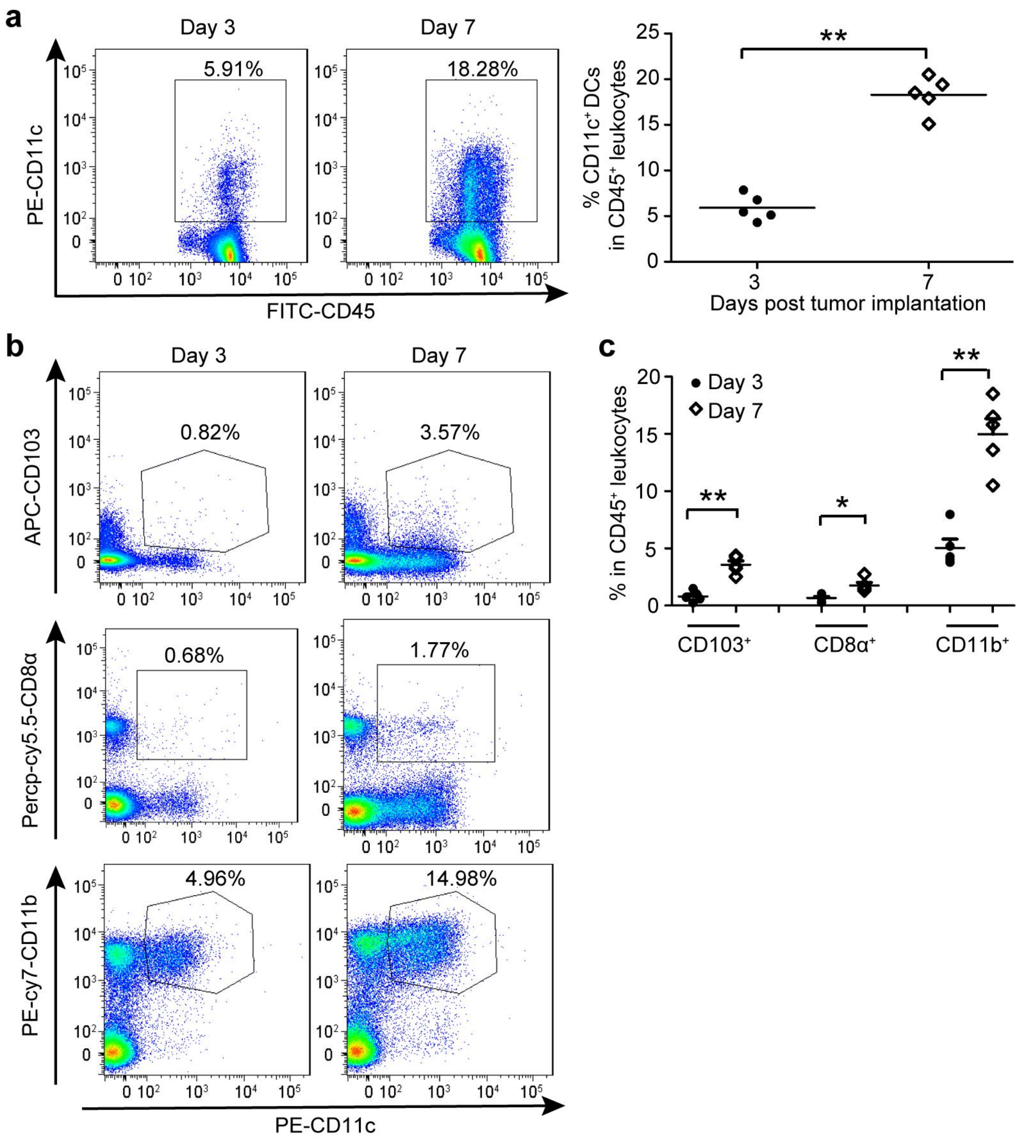


**Supplementary Figure 6**. Analysis of DCs in blood of orthotopic HCC mice treated with Flt3L. Flt3L (800 μg/kg/day) was injected into day-3 orthotopic HCC mice subcutaneously for 8 days consecutively and blood was collected at day 3 and day 7 after tumor inoculation. Flow cytometric and quantitative analysis of CD11c$^{+}$ (**a**) and CD103$^{+}$CD11c$^{+}$, CD8α$^{+}$CD11c$^{+}$ and CD11b$^{+}$CD11c$^{+}$ (**b** and **c**) DCs in blood of orthotopic HCC mice on day 3 and 7 after tumor inoculation (n=5; two-tailed t test). *p<0.05, **p<0.001; n.s means not significant.

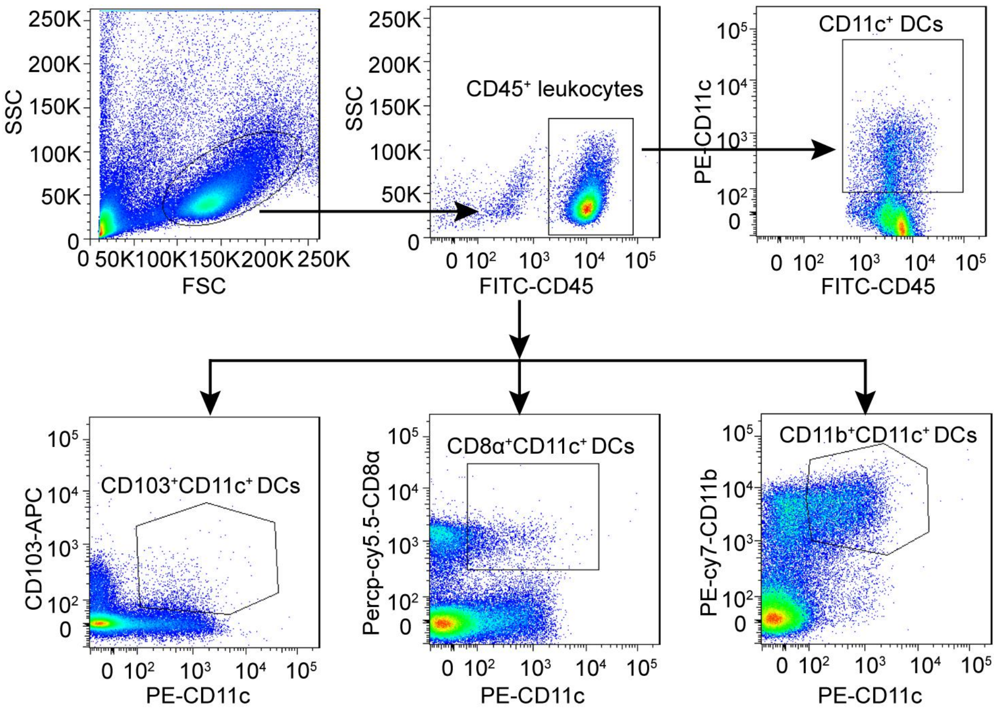


**Supplementary Figure 7**. Representative gating information for the flow cytometic analysis of different DC subsets in mouse peripheral blood.

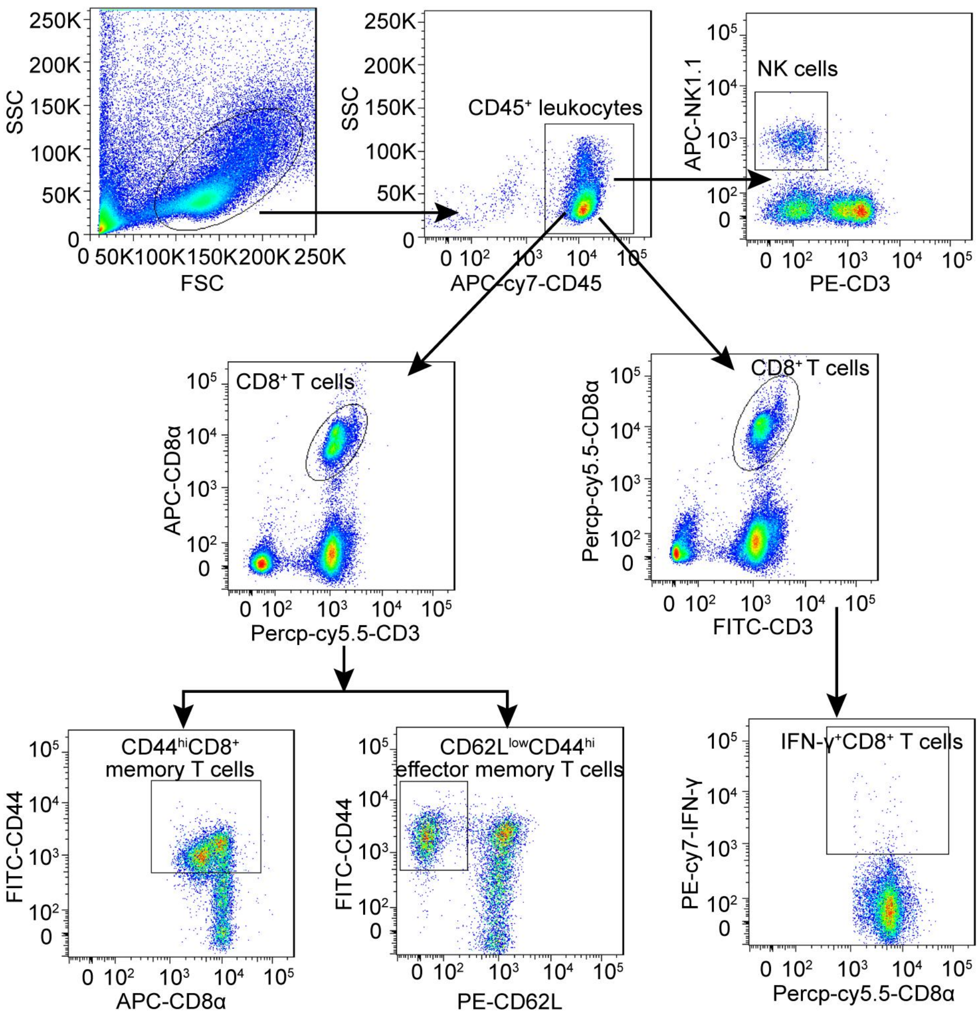


**Supplementary Figure 8**. Representative gating information for the flow cytometric analysis of different lymphocytes, effector and memory T cells in mouse peripheral blood.

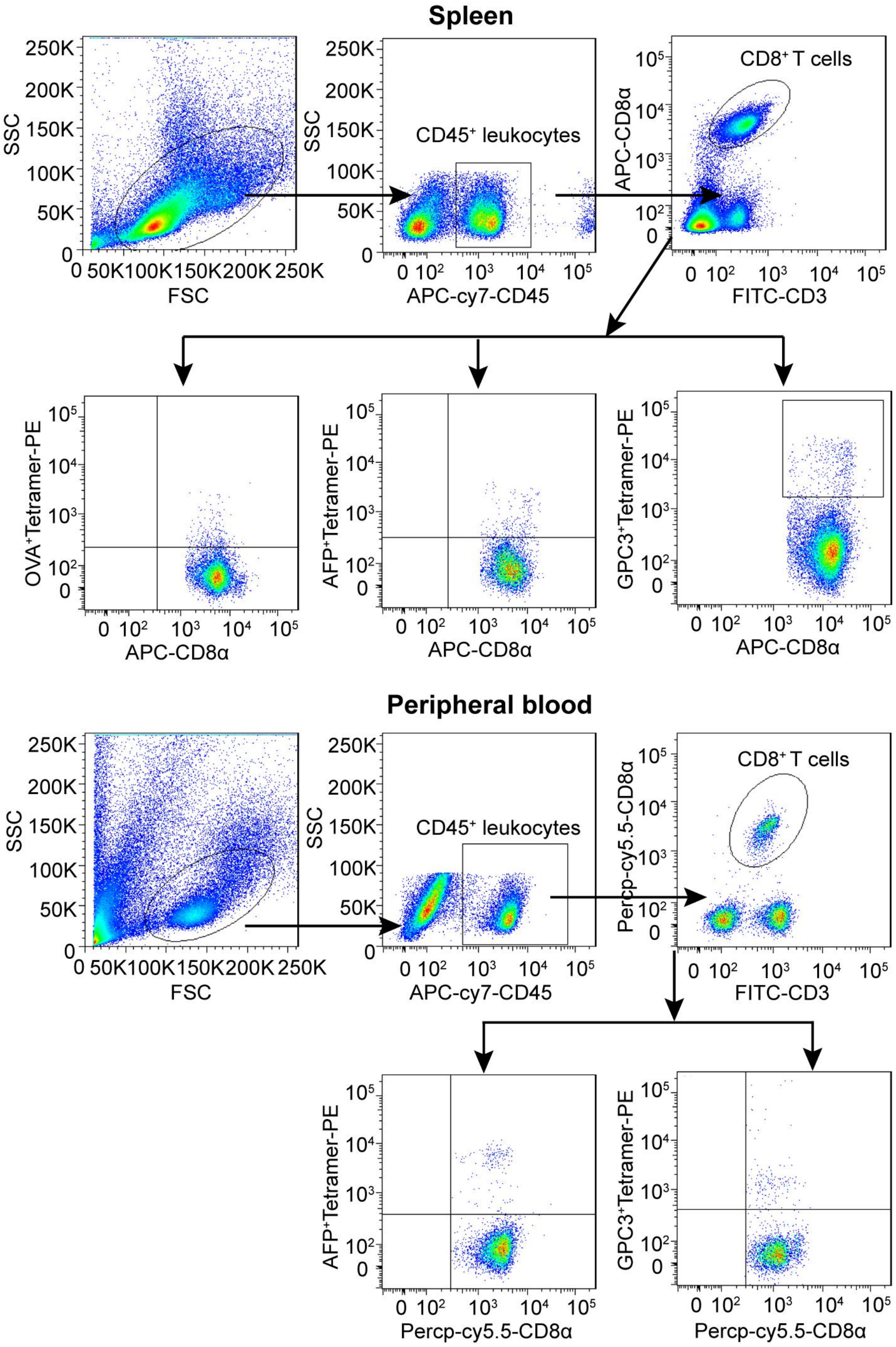


**Supplementary Figure 9**. Representative gating information for the flow cytometric analysis of different antigen-specific T cells in mouse spleen and peripheral blood.

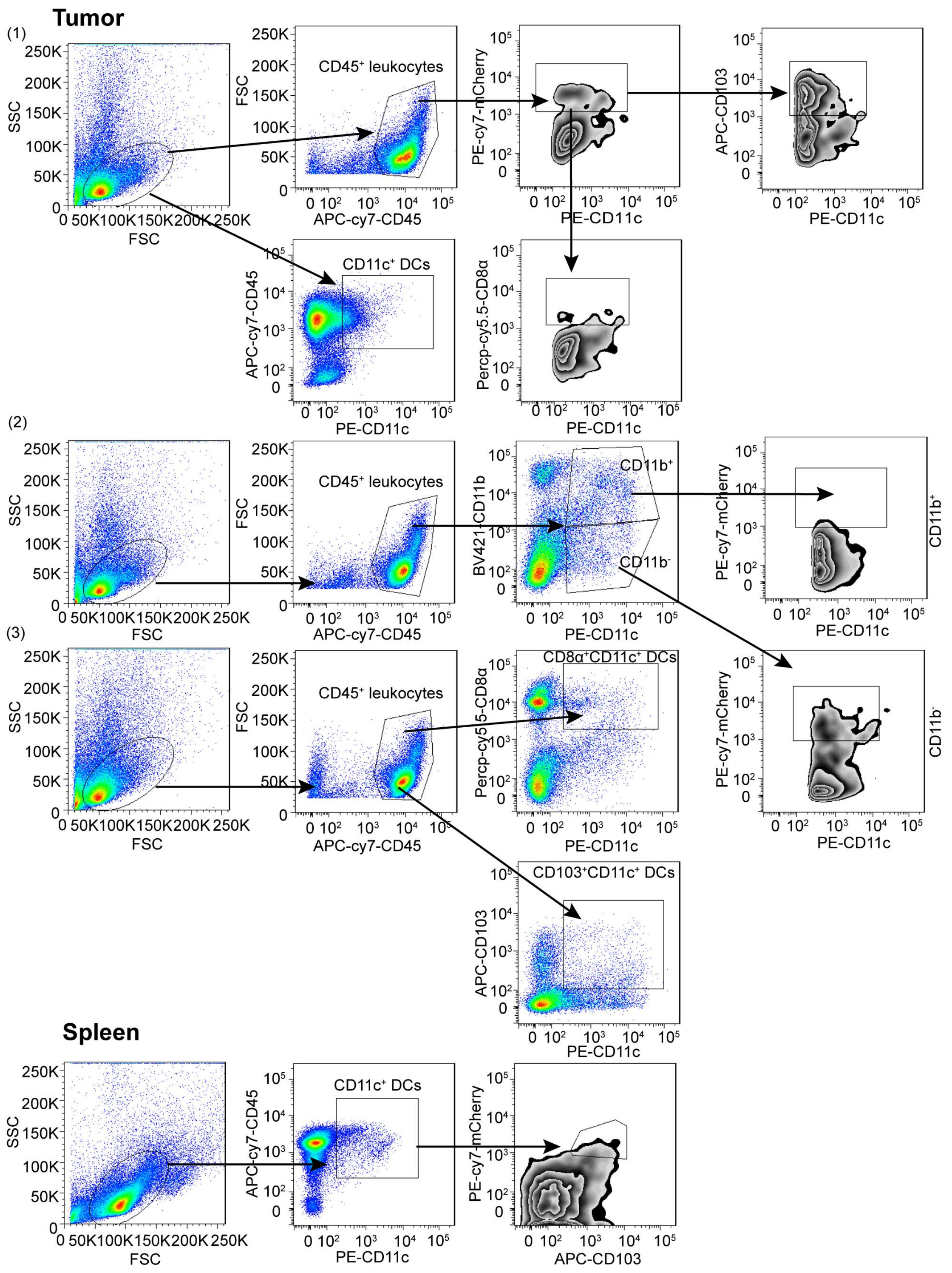


**Supplementary Figure 10**. Representative gating information for the flow cytometric analysis of different DC or mCherry+ DC subsets in tumor and spleen. (1)-(3) represents different DC or mCherry+ DC subsets in tumor.

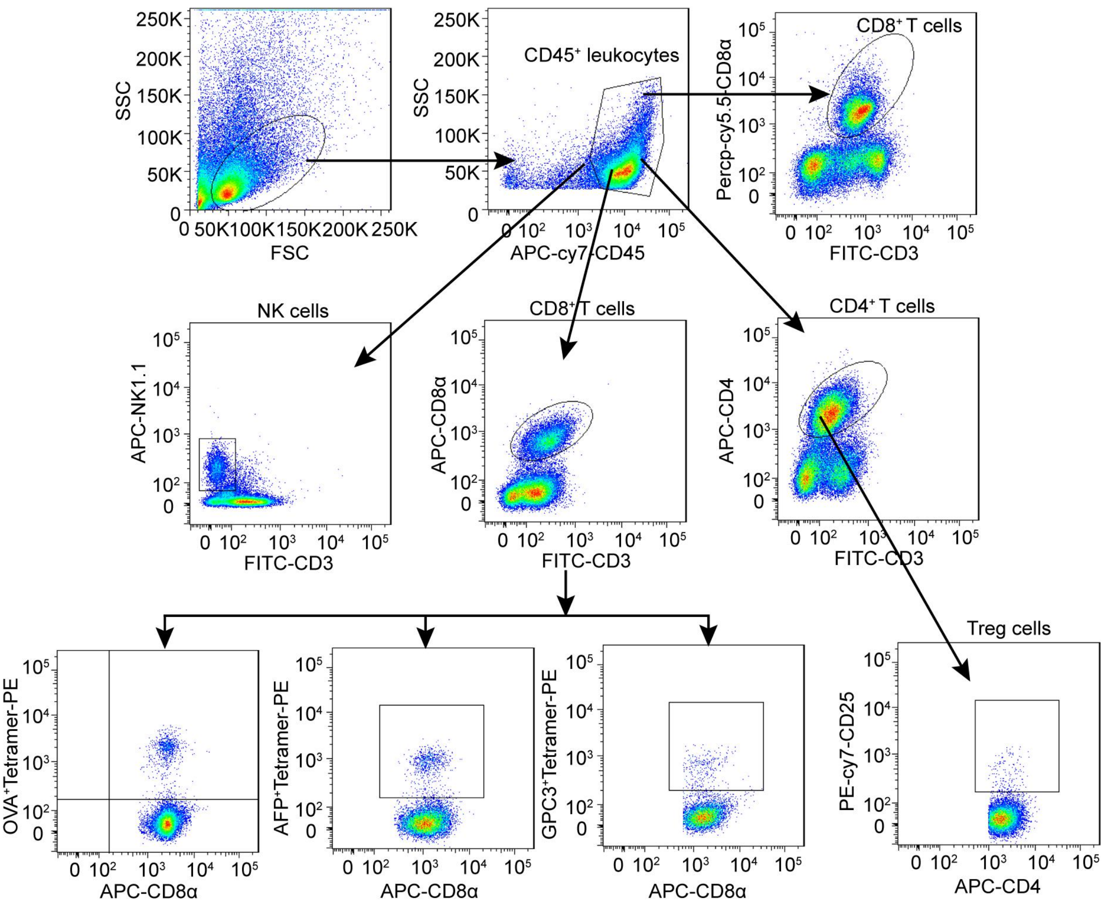


**Supplementary Figure 11**. Representative gating information for the flow cytometric analysis of different lymphocytes, Tregs and antigen-specific T cells in tumor.
